# Temperature dependent chemical shifts of pyruvate and lactate enable in vivo hyperpolarized ^13^C MRSI thermometry

**DOI:** 10.1038/s44303-025-00081-3

**Published:** 2025-05-06

**Authors:** Wolfgang Gottwald, Luca Nagel, Jason G. Skinner, Martin Grashei, Sandra Sühnel, Nadine Setzer, Wolfgang Eisenreich, Mary A. McLean, Ferdia A. Gallagher, Jae Mo Park, Zumrud Ahmadova, Martin Gierse, Senay Karaali, Stephan Knecht, Ilai Schwartz, Irina Heid, Geoffrey J. Topping, Frits H. A. van Heijster, Franz Schilling

**Affiliations:** 1 Department of Nuclear Medicine, TUM School of Medicine and Health, TUM University Hospital, Technical University of Munich, Munich, Germany; 2 Bavarian NMR Center (BNMRZ), Structural Membrane Biochemistry, TUM School of Natural Sciences, Technical University of Munich, Garching bei München, Germany; 3 Cancer Research UK Cambridge Centre, University of Cambridge, Cambridge, UK; 4 Department of Radiology, University of Cambridge, Cambridge, UK; 5 Advanced Imaging Research Center, University of Texas Southwestern Medical Center, Dallas, TX USA; 6 Department of Radiology, University of Texas Southwestern Medical Center, Dallas, TX USA; 7 Department of Biomedical Engineering, University of Texas Southwestern Medical Center, Dallas, TX USA; 8 NVision Imaging Technologies GmbH, Ulm, Germany; 9 Institute of Diagnostic and Interventional Radiology, TUM School of Medicine and Health, TUM University Hospital, Technical University of Munich, Munich, Germany; 10 Munich Institute of Biomedical Engineering, Technical University of Munich, Garching, Germany; 11 German Cancer Consortium (DKTK), Partner Site Munich and German Cancer Research Center (DKFZ), Heidelberg, Germany

**Keywords:** NMR spectroscopy, Imaging techniques, NMR spectroscopy

## Abstract

The chemical shift of many molecules changes with temperature, which enables non-invasive magnetic resonance imaging (MRI) thermometry. Hyperpolarization methods increase the inherently low ^13^C MR signal. The commonly-used hyperpolarized probe [1-^13^C]pyruvate, and its metabolic product [1-^13^C]lactate, exhibit temperature and concentration dependent chemical shift changes that have not previously been reported. These effects were characterized at 7 T and 11.7 T in vitro and applied for in vivo thermometry both preclinically at 7 T and to human data at 3 T. Apparent temperature values from mouse abdomen and brain were similar to rectally measured temperature. Human brain and kidney apparent temperatures from ^13^C MRSI were lower than known physiological temperatures, suggesting that additional effects may currently limit the use of this method for determining absolute temperature in humans. The temperature dependent chemical shift changes also have implications for sequence design and for in vitro studies with hyperpolarized pyruvate.

## Introduction

In all living systems, temperature is a critical component of homeostasis. Changes in body temperature were already recognized to be related to disease more than 2000 years ago by Hippocrates^1,2^. Since the first temperature measurements by Galileo, methods for temperature measurements have expanded beyond the classical thermometer^3^. During the development of magnetic resonance imaging (MRI), it was found that the resonant frequency of protons in many molecules depends on the temperature of their environment, enabling non-invasive temperature measurements in living organisms^4–7^. MR thermometry has both diagnostic and therapeutic applications and has been used clinically^7–15^. Most prominently, the temperature-dependence of the water proton resonance frequency (-0.01 ppm/°C)^5,7,11,16,17^ has been used to measure relative temperature in vivo without use of contrast agents. Other MR thermometry methods make use of ^19^F, hyperpolarized ^129^Xe, T_1_, T_2_, diffusion, magnetization transfer or contrast agents^8,18–20^.

Hyperpolarized (HP) ^13^C MRI has evolved as a valuable method^21^ to non-invasively measure metabolic activity^22,23^, pH^24–26^, and perfusion^24,27^. Most commonly, a solution containing HP [1-^13^C]pyruvate (PA) is injected intravenously. The hyperpolarized ^13^C label is exchanged with the larger endogenous lactate pool in the reaction catalysed by lactate dehydrogenase (LDH) enabling measurements of metabolic flux^28^. Since tumors exhibit an increase of glycolytic activity, together with elevated lactate production in the presence of oxygen (Warburg effect^29^), there is an increasing research interest in studying cancer using HP PA^30–34^. Hyperpolarized ^13^C MRI using dissolution dynamic nuclear polarization (d-DNP) has been translated from a preclinical setting towards patients in over 60 studies^33,35^. Lately, other hyperpolarization methods such as parahydrogen-induced polarization (PHIP)^36–39^ and signal amplification by reversible exchange (SABRE)^40,41^ have gained interest and are currently employed in preclinical settings.

In this work, the temperature and concentration dependent chemical shift of the [1-^13^C]lactate (LA) and [1-^13^C]pyruvate resonant frequencies are investigated. We characterize the dependencies at two magnetic fields (7 T and 11.7 T), temperatures ranging from 18 to 42 °C, metabolite concentrations of 5–600 mM and in aqueous solutions and whole blood. To investigate the translatability from a controlled phantom setting to living organisms, a preclinical study in healthy mice was conducted. Each animal was injected twice within one experimental session using HP PA, first at an elevated body temperature^42^ of 38 °C and then at a lowered temperature (31 or 34 °C). Additionally, we retrospectively analyze data from three published human brain HP [1-^13^C]pyruvate magnetic resonance spectroscopic imaging (MRSI) studies using our novel thermometry method, which investigated healthy brain metabolism, kidney metabolism and glioblastoma (GBM)^30,43–45^.

## Results

### Thermally polarized calibration measurements

**Fig. 1:**
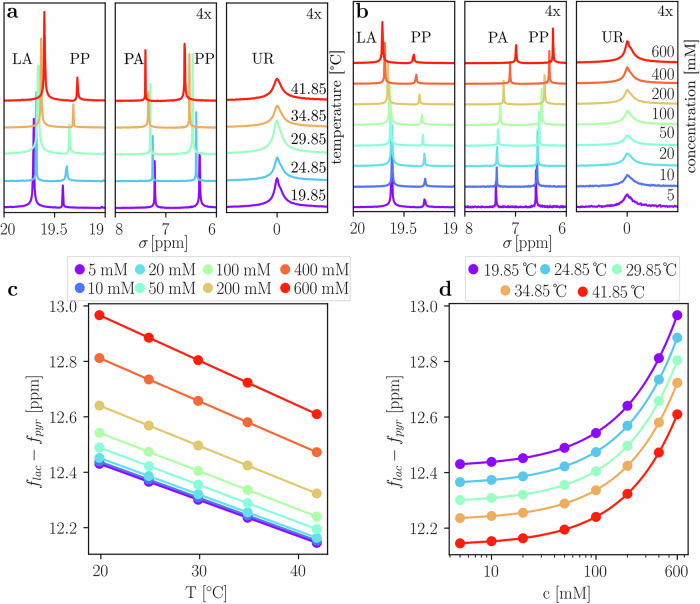
Temperature dependency of ^13^C-labeled pyruvate and lactate in phantom calibration experiments at 11.7 T. **a** Spectra of aqueous solutions containing the ^13^C-labeled molecules [1-^13^C]pyruvate (PA), [1-^13^C]lactate (LA) and [^13^C]urea (UR) at 50 mM concentration each. Measurements at different 5 temperatures show their temperature dependent chemical shifts, with respect to a reference ([^13^C]urea, UR). During the sample preparation process, [1-^13^C]parapyruvate (PP) was also formed, due to the acidic environment of the original solution before titration^61^ (pH 2.3). **b** Eight different solutions, ranging from 5 mM to 600 mM ^13^C at 34.85 °C, highlighting the concentration-dependent chemical shifts of pyruvate, lactate and parapyruvate. Urea and pyruvate spectra subplots (**a**, **b**) are multiplied by a factor of 4 for better visibility. **c** Relative chemical shift between pyruvate and lactate for 5 different temperatures, fitted to a linear regression model. **d** Relative chemical shift between pyruvate and lactate for 8 different concentrations, fitted to a power function, as described in Table S4 and equation S2. Temperatures were set in Kelvin on the spectrometer and converted to Celsius for comparability with in vivo data.

The temperature-dependent chemical shift changes of [1-^13^C]pyruvate and [1-^13^C]lactate with reference to [^13^C]urea (UR) were characterized in vitro in aqueous solution at five temperatures at 11.7 T ranging from 20 to 42 °C, resulting in the ^13^C spectra shown in Fig. 1a–c. Signals from lactate, pyruvate, urea and two peaks from parapyruvate (PP) were detected. The chemical shift difference between lactate and pyruvate, at a concentration of 5 mM, decreases by 0.013 ± 0.002 ppm/°C (R^2^ = 0.99, see Table 1) with increasing temperature. The two PP peaks shift in the same direction as pyruvate and lactate, respectively. Spectra shown in Fig. 1 have been referenced to urea. During initial experiments, a concentration-dependent chemical shift change between pyruvate and lactate was also observed, which is characterized in Fig. 1b–d for eight concentrations ranging from 5 to 600 mM (pH = 7.73 ± 0.35). At higher concentrations, lactate and pyruvate shift away from each other. However, at the expected low in vivo concentrations of the metabolites (below 10 mM), this effect is negligible compared to the magnitude of temperature dependent shifts.

**Table 1:** Fit parameters of linear regression models shown in Fig. 2

	HP LDH 7 T	HP Blood 7 T	5 mM 11.7 T	100 mM 11.7 T
pitch [ppm/°C]	−0.01473 ± 0.00014	−0.01301 ± 0.00050	−0.01297 ± 0.00002	−0.01373 ± 0.00007
crossing [°C]	12.781 ± 0.005	12.737 ± 0.015	12.688 ± 0.006	12.815 ± 0.021

Parameters for all concentrations are found in Supplemental Information, Table S3.

### Hyperpolarized in vitro spectroscopy

**Fig. 2:**
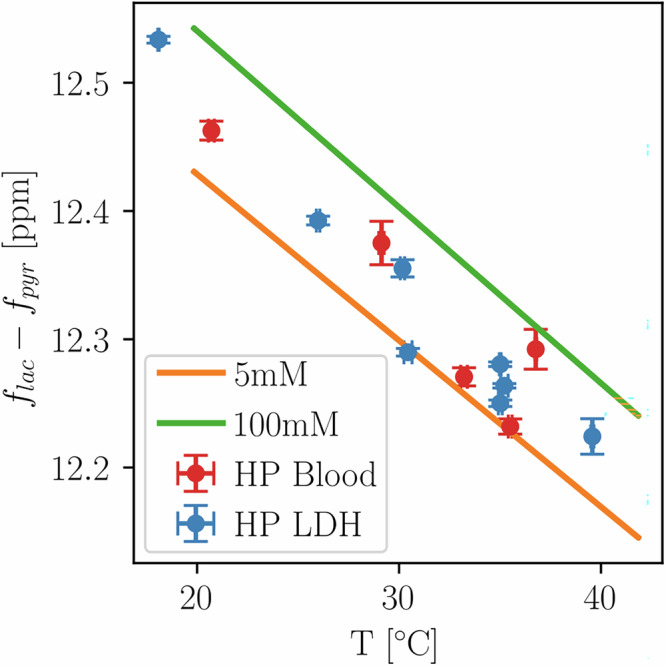
Temperature dependency of ^13^C-labeled pyruvate and lactate for hyperpolarized phantom experiments at 7 T compared to thermal calibrations at 11.7 T. Blood data points (red) show frequency difference of co-polarized [1-^13^C]pyruvate and [1-^13^C]lactate mixed with rodent blood that was preheated to five different temperatures (20–36 °C). For LDH data points, HP [1-^13^C]pyruvate was injected into a buffered solution containing LDH and nicotinamide adenine dinucleotide (NADH) to facilitate lactate production at nine different temperatures ranging from 18–40 °C. Both HP experiments were conducted at in vivo concentrations of 8 mM pyruvate and a linear regression model (not shown) was fit to both datasets. Line plots (5 mM and 100 mM from 11.7 T experiments) show linear regression models to data presented in Fig. 1d for two exemplary concentrations to demonstrate similarity between measurements at 7 T and 11.7 T and different media. Corresponding fit parameters and uncertainties are displayed in Table 1, confidence intervals for 5 mM and 100 mM linear regression fits are too small to depict.

HP experiments at 7 T using LDH phantoms are compared to thermal measurements in Fig. 2 (pH = 8.5 ± 0.2) and HP blood results are plotted in red (pH = 7.0 ± 0.2). Linear calibration fits to phantom data show similar trends, with deviations in blood and LDH experiments due to worse temperature control and concentration effects. Linear fit parameters from calibrations plotted in Fig. 2 are shown in Table 1, with all measurements shown in the Table S3. Using the linear dependency relationship, absolute temperatures can be calculated from chemical shift differences between lactate and pyruvate for given concentrations. Similarly, the concentration for a fixed temperature can be computed from the concentration fit functions given in Table S4 derived from fits to equation S2.

### Healthy mouse abdomen apparent temperature mapping using HP pyruvate

**Fig. 3:**
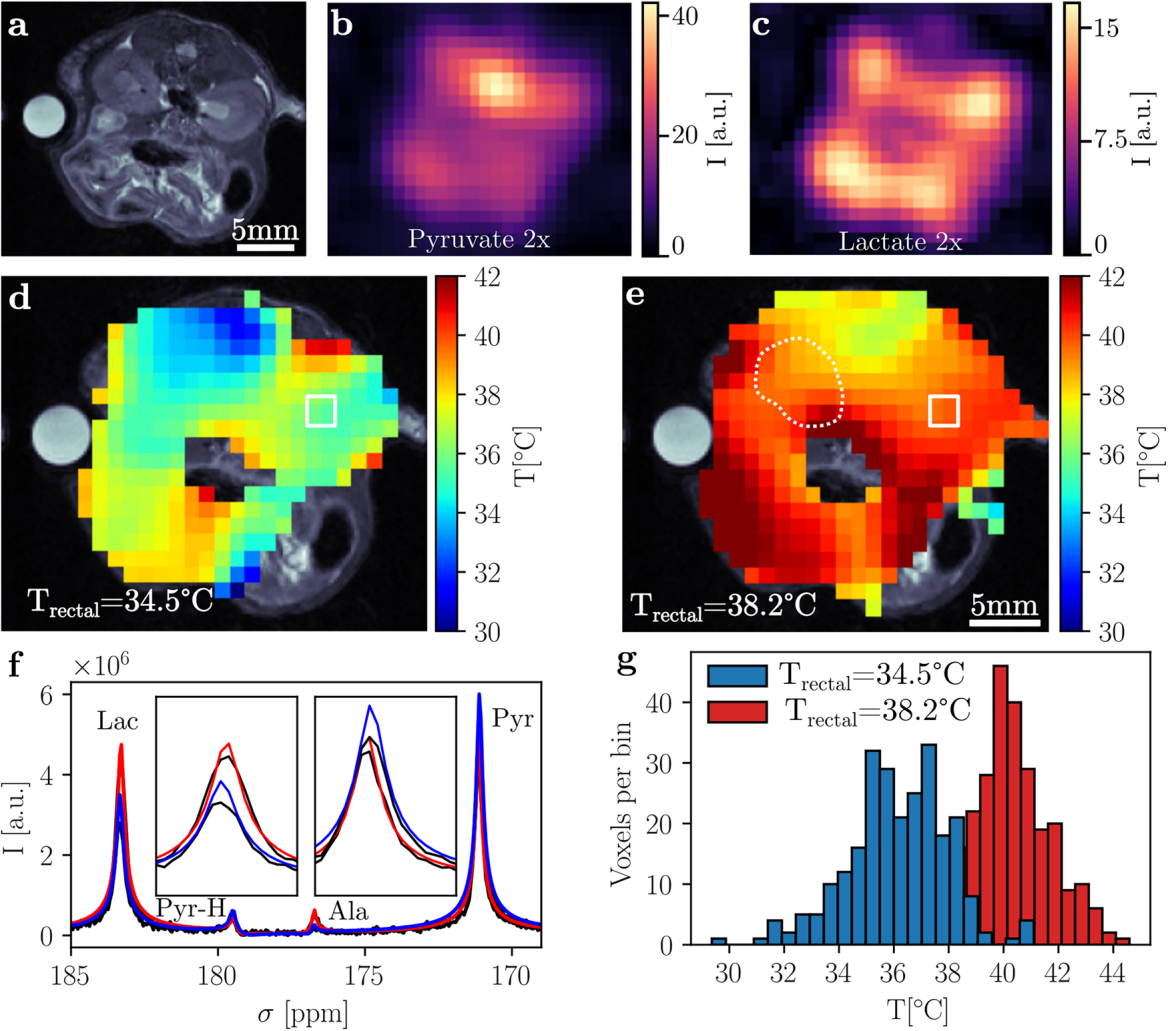
In vivo 2D FID-CSI data obtained from 2 injections of an exemplary mouse with HP PA at 38.2 °C and 34.5 °C rectal body temperatures. T_2_w anatomical reference image (**a**) and both pyruvate (**b**) and lactate (**c**) peak area integral maps from the CSI at a decreased rectal temperature. **d** 2x-interpolated apparent temperature map (average temperature: 36.1 ± 1.8 °C, native resolution shown as white square) calculated from CSI data for the injection at a decreased rectal temperature. **e** Elevated rectal temperature map (average temperature: 40.0 ± 1.7 °C, kidney ROI shown as white dotted line). **f** Exemplary spectrum (black) and fit of a kidney voxel (red: 38.2 °C, blue: 34.5 °C, white squares in **d**/**e**) for both elevated and decreased rectal temperature injections, with pyruvate and lactate peak areas as insets. **g** Histogram of apparent temperatures for both datasets shown in (**d**, **e**).

In vivo 2D free induction decay chemical shift imaging sequence (FID-CSI) from an exemplary healthy animal (cohort 1) shows a clear difference of apparent temperature for mean slice values (single slice placed over the kidneys in axial orientation (see Fig. 3a–c for anatomical reference, pyruvate and lactate intensity maps), mean over all voxels with a fit error below 0.5 °C within the slice) between the first injection at 38.2 °C rectal temperature and the second injection at 34.5 °C (see apparent temperature maps in Fig. 3d/e, exemplary spectrum in Fig. 3f and corresponding histograms in Fig. 3g). While the absolute values of abdominal apparent temperature from CSI (T_CSI_) are higher than rectal temperature (T_rec_) as measured by a PT-100 probe, the relative changes in apparent temperature for these two measurements are the same within error bounds (T_CSI_ = 40.0 ± 1.7 °C and 36.1 ± 1.8 °C vs. T_rec_ = 38.2 ± 0.1 °C and 34.5 ± 0.1 °C, (i.e.,: ΔT_CSI_ = 3.9 °C, vs. ΔT_rec_ = 3.7 °C).

**Fig. 4:**
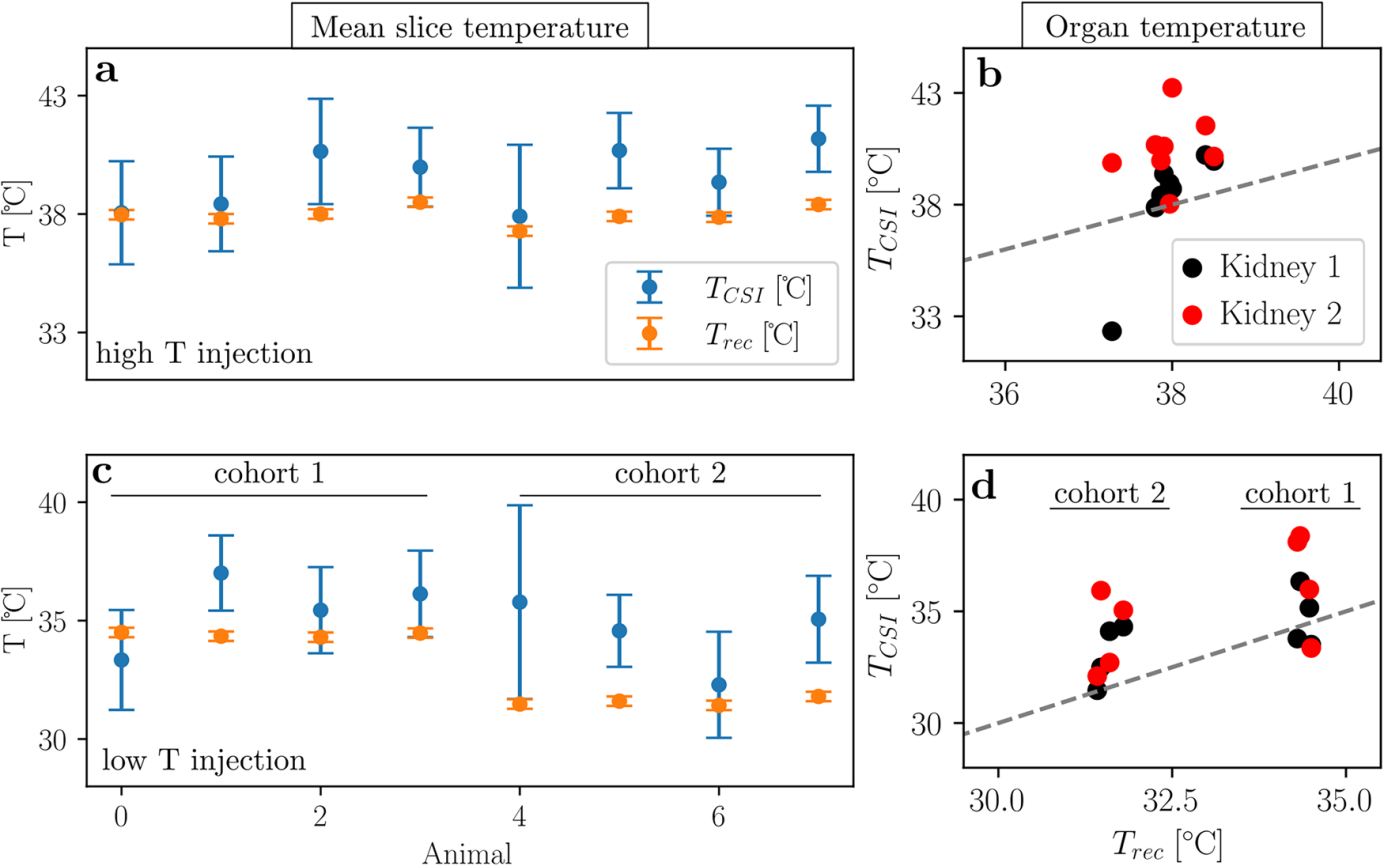
Temperature mapping using HP PA and CSI in eight animals. **a**, **c** Average slice apparent temperature for the injection at an elevated rectal temperature (38.0 ± 0.3 °C, *n* = 8) as well as the following injection at decreased rectal temperature (34.5 ± 0.1 °C, 31.6 ± 0.1 °C, *n* = 4 each). Error bars are one standard deviation obtained from the fit-error thresholded temperature map, as shown in Fig. 3d–f. Apparent temperatures calculated from CSI (T_CSI_) are on average higher than rectal temperature (T_rec_). **b**, **d** Average kidney apparent temperatures obtained from segmentation in the anatomical image and application of that mask to the temperature map plotted against rectal temperature. The bisectrix is drawn for clarification.

Comparing data from all eight animals, differences between the injections at elevated and decreased rectal temperature measurements become apparent (Fig. 4 and Table 2).

**Table 2:** Rectal temperature compared to abdominal and kidney apparent temperature computed from ^13^C CSI

Cohort	T_rec_ [°C]	T_CSI_ [°C]	T_kidney_ [°C]
1 and 2 (*n* = 8)	38.0 ± 0.4	39.5 ± 1.2 (*p* = 0.005)	39.4 ± 1.5 (*p* = 0.02)
1 (*n* = 4)	34.4 ± 0.1	35.5 ± 1.4 (*p* = 0.28)	35.6 ± 1.4 (*p* = 0.26)
2 (*n* = 4)	31.6 ± 0.1	34.4 ± 1.3 (*p* = 0.03)	33.5 ± 1.1 (*p* = 0.04)

*p*-values were computed using a paired t-test (scipy.stats.ttest_rel) to assess whether differences are significant between rectal temperature vs. abdominal slice (T_CSI_) or kidney temperature (T_Kidney_) for both cohorts together (first row) and each cohort separately (second and third row).

For the elevated temperature measurements, T_CSI_ agrees with T_rec_ for 4 of 8 animals within error bounds (Fig. 4a). For the decreased rectal temperature scans, the two cohorts need to be distinguished (cohort 1 at T_rec_ = 34.4 °C and cohort 2 at T_rec_ = 31.6 °C). For the former, the abdominal apparent temperature from CSI is within the error bounds of the rectal temperature in 2 out of 4 animals (Fig. 4c). For the latter, T_CSI_ appears to be increased with respect to T_rec_ in 3 out of 4 animals (Fig. 4c). Relative changes in apparent temperature vs. rectal temperature are similar (cohort 1: ΔT_CSI_ = 3.8 ± 1.7 °C vs. ΔT_rec_ = 3.7 ± 0.3 °C, *p* = 0.88, cohort 2: ΔT_CSI_ = 5.4 ± 2.2 °C vs. ΔT_rec_ = 6.3 ± 0.3 °C, *p* = 0.40). To assess intra-slice variability, kidneys were segmented and mean apparent temperatures of both organs compared to respective rectal temperatures (Fig. 4b, d). For measurements at elevated rectal temperature and for mice in cohort 1 imaged at a rectal temperature of 34.4 °C, kidney apparent temperature agrees with rectal temperature within error margins (Fig. 4b, d). Similarly to whole abdominal apparent temperature (Fig. 4c), animals in cohort 2, imaged at 31.6 °C, appear to show an increased kidney apparent temperature compared to their rectal temperatures (Fig. 4d).

### Differences in metabolism at lower temperatures

**Fig. 5:**
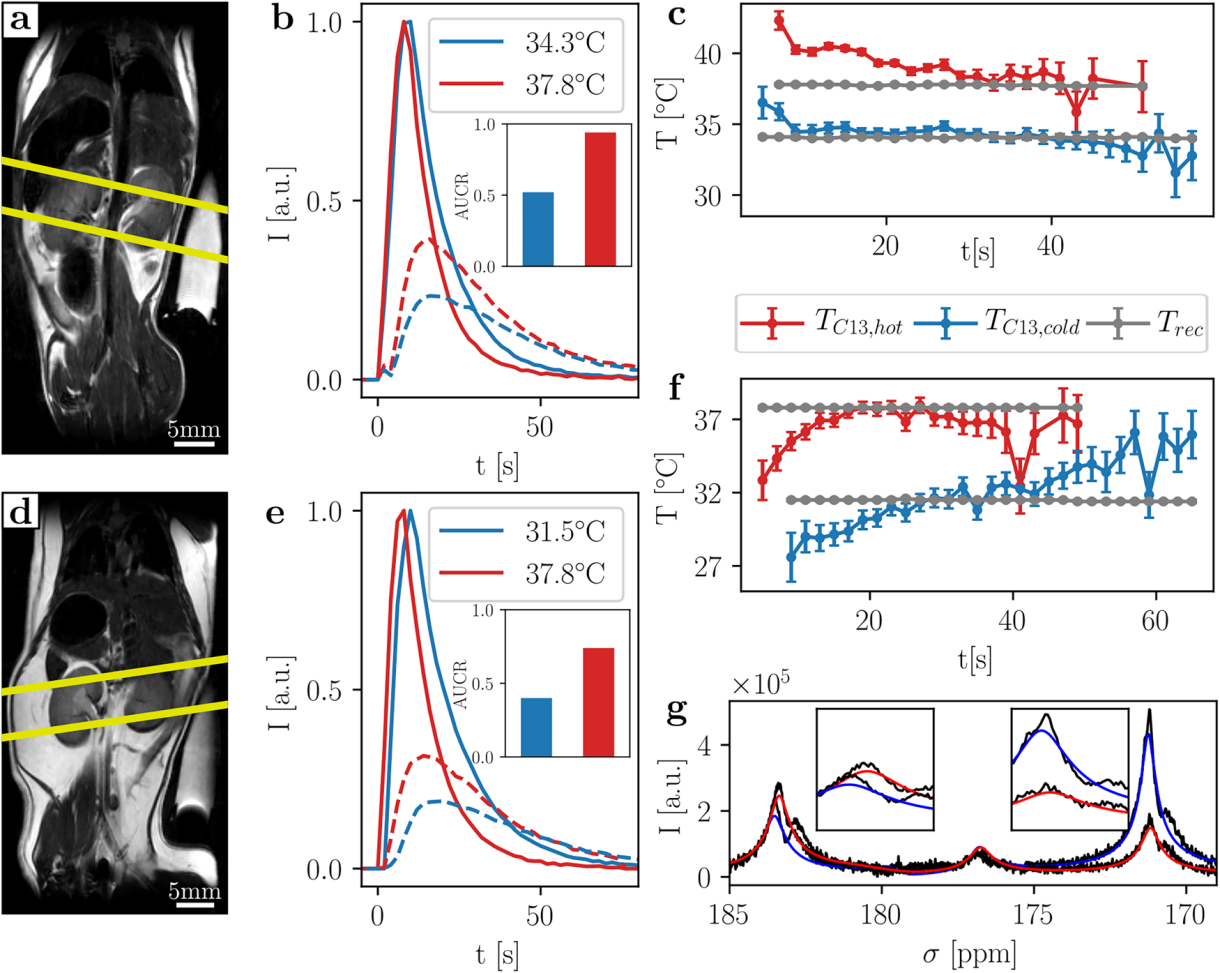
In vivo slice selective MR spectroscopy in two animals at temperatures 38–34 °C and 38–31 °C. **a**, **d** T_2_w anatomical reference images with slice placement over kidneys (yellow lines). **b**, **e** Time domain fit FID magnitude amplitude curves for pyruvate (solid) and lactate (dashed) for two successive injections at elevated and decreased rectal temperatures in the same animals. AUCR decreases with decreased rectal temperature (Animal 1: 37.8/34.3 °C: AUCR = 0.94/0.52, Animal 2: 37.9/31.5 °C: AUCR = 0.74/0.40, while mean apparent temperature calculated from spectra is slightly higher than the rectal temperature (T_C13,high1_ = 39.0 ± 1.3 °C, T_C13,low1_ = 34.2 ± 0.9 °C, T_C13,high2_ = 36.4 ± 1.4 °C, T_C13,low2_ = 32.0 ± 2.2 °C). **c**, **f** MR-measured mid-abdominal / kidney apparent temperature drifts over time after injection for repetitions with high SNR in both pyruvate and lactate channels as well as the effectively stable recorded rectal temperature. **g** Exemplary spectra and fits for both injections in (**e**) at approximate time of lactate peak with pyruvate and lactate peak areas as inserts.

Dynamic slice selective spectroscopy (Fig. 5, slice placement over kidneys as seen in Fig. 5a, b) showed a 44 ± 1% and 46 ± 1% decrease in lactate production at lower rectal temperatures (area under the curve ratio (AUCR) = 0.94/0.52 at 37.8 °C/34.3 °C and AUCR = 0.74/0.40 at 37.8 °C/31.5 °C, Fig. 5b, e). Absolute apparent temperatures computed are slightly higher than the rectal measurements and also vary over time (T_C13,high1_ = 39.0 ± 1.3 °C and T_C13,high2_ = 36.4 ± 1.4 °C vs. T_rec,high1_ = 37.8 ± 0.1 °C, T_C13,low1_ = 34.2 ± 0.9 °C vs T_rec,low1_ = 34.3 ± 0.1 °C, T_C13,low2_ = 32.0 ± 2.2 °C vs T_rec,low2_ = 31.5 ± 0.1 °C, Fig. 5c, f). This effect is especially pronounced in the animal injected at 31.5 °C (Fig. 5f), where the apparent temperature increases after injection. For the warmer injection, the apparent temperature approaches the rectal temperature after 15 s, while, for the colder injection, it continues increasing, consistent with the core temperature being maintained higher than the directly measured rectal temperature. The difference in AUCR for hot and cold measurements is consistent with reduced activity of LDH at lower temperatures^46^, which results in lower lactate production. Other contributing effects to this difference, such as perfusion changes or temperature of the injected bolus, cannot be excluded but are difficult to isolate as contributing factors. Similar results (decrease by 55% and 75% in AUCR) were acquired for two animals imaged using a 3D-bSSFP sequence at elevated and decreased rectal temperatures (Figure S1).

### Healthy mouse brain apparent temperature

Whole brain apparent temperature from two animals scanned twice each correlates well with monitored rectal temperature (T_CSI_ = 38.2 ± 2.4 °C and T_rec_ = 37.8 ± 0.3 °C, *n* = 4). Brain apparent temperature maps are shown in Figure S9 and S10.

### Apparent temperature from human ^13^C MRS in the brain and abdomen

Using calibration functions for 5 mM metabolite concentration, brain apparent temperature maps from nine healthy volunteers (data published by Kaggie et al.^43^) were generated, with an example given in Figure S3 (apparent temperature maps and mean slice apparent temperatures from all volunteers are found in Figures S4 and S6 and Table S5). Pyruvate signal per patient as a perfusion-weighted quantity was plotted against apparent temperature resulting in no systematic correlation (Figure S5). Similarly, mean apparent brain temperature values were generated from 7 injections of hyperpolarized [1-^13^C]pyruvate into four healthy volunteers (data published by Ma et al.^44^, all values shown in Figure S6 and Table S7).

**Fig. 6:**
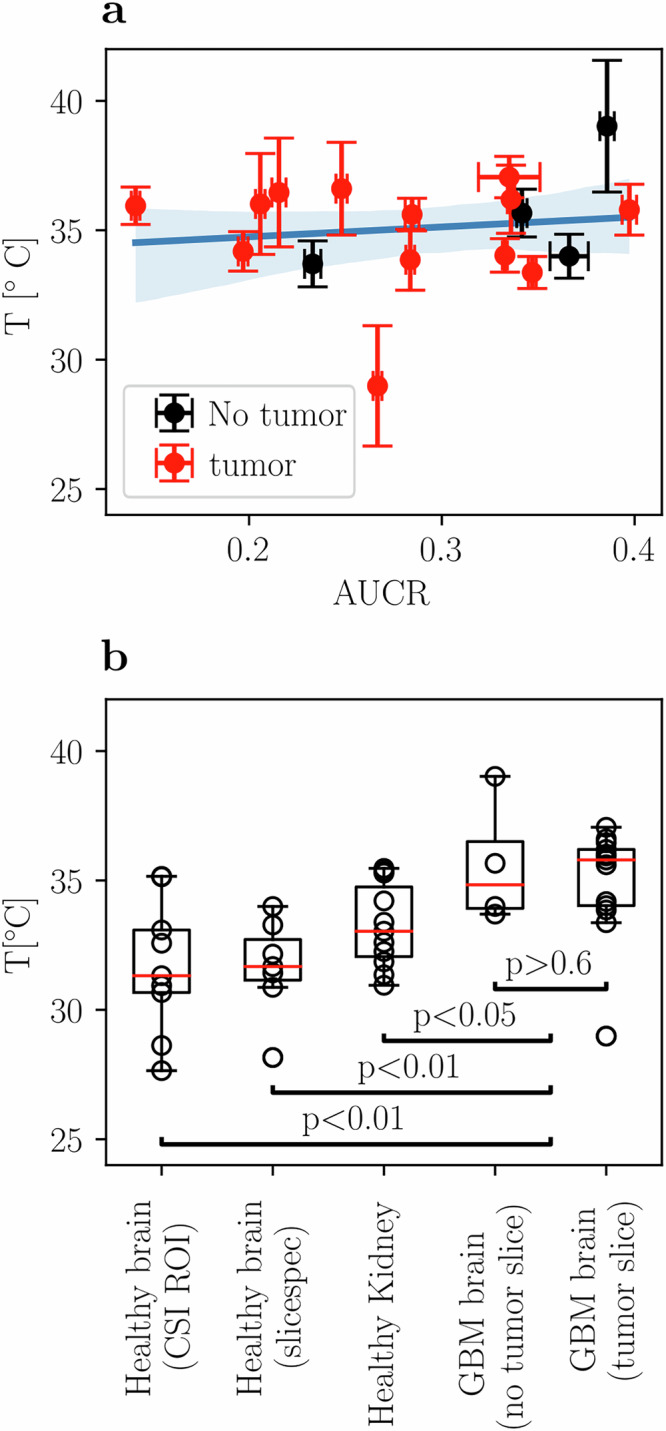
Human glioblastoma metabolism versus apparent temperature from ^13^C-spectroscopy. **a** Slice-selective spectroscopy data from patients with GBM tumors in three slices each (total 18 slices with fit accuracy of 3 °C or better as well as SNR above 5). No correlation between AUCR and apparent temperature could be estimated (R^2^ = 0.01, slope = 3.8 ± 7.5 °C^-1^), nor any difference in apparent temperature between slices containing a tumor and tumor-free slices (*p* > 0.6). **b** Comparing apparent temperatures from healthy human brain CSI datasets (Healthy 1^43^: T = 31.7 ± 2.5 °C, *n* = 9), healthy brain slice selective spectroscopy (Healthy 2^44^: T = 31.7 ± 1.9 °C, n = 7) and GBM slice selective spectroscopy (GBM^30^: T = 35.1 ± 2.1 °C, *n* *=* *6*), shows an increase in temperature for tumor patients (*p* < 0.01). Healthy human kidney apparent temperature is below physiological values (T = 33.3 ± 1.6 °C, *n* = 11) and, when compared to GBM apparent temperature, a significant difference is found (*p* < 0.05).

Mean apparent temperature values computed from slice selective ^13^C spectroscopy in GBM patients (data published by Zaccagna et al.^30^, all values shown in Figure S6 and Table S6) are higher than those of healthy volunteers (Healthy 1^43^: T = 31.7 ± 2.5 °C (*n* = 9), Healthy 2^44^: T = 31.7 ± 1.7 °C (*n* = 7) vs. GBM^30^: T = 35.1 ± 2.1 °C (*n* = 6), Fig. 6b). For glioblastoma, an increase in metabolic activity in the whole brain has been reported^30^, which we hypothesized could also lead to a localized increase in temperature. However, no correlation between AUCR and brain temperature was found (Fig. 6a, R^2^ = 0.01, slope = 3.8 ± 7.5 °C^-1^).

Finally, human abdominal slice-selective MRS data from 12 volunteers scanned a total of 14 times was analyzed (Fig. 6b, all data shown in Figure S6 and Table S8). Slice mean values are below physiological values but slightly higher than brain apparent temperature (Renal^45^: 33.3 ± 1.6 °C (*n* = 10)).

## Discussion

For the first time, the temperature dependent chemical shifts of [1-^13^C]pyruvate and [1-^13^C]lactate have been reported and used for in vivo temperature sensing in addition to acquiring metabolic information. The relative chemical shift difference between pyruvate and lactate at 18–42 °C was characterized in aqueous and blood solutions and at concentrations ranging from 5 mM to 600 mM of ^13^C-labeled molecules. We found a linear temperature-dependent chemical shift of 0.013 ppm/°C, where pyruvate and lactate peaks diverge at lower temperatures.

We assessed the dependency of the chemical shifts of [1-^13^C]pyruvate and [1-^13^C]lactate on pH as well using published data^47,48^, and found that lactate shifts in the relevant in vivo pH range by maximally 0.006 ppm, while pyruvate does not exhibit a relevant shift in this range (see Supplemental Information, Figure S8). Therefore, the pH related uncertainty is on the order of ±0.5 °C. Dependence of blood oxygenation on [1-^13^C]pyruvate and [1-^13^C]lactate chemical shifts was assessed to be of minor concern (Supplemental Information, Figure S7).

Mouse abdominal CSI-derived apparent temperature was higher compared to simultaneously acquired rectal probe temperatures (Fig. 4), which is unlikely to be only due to a variation between core and rectal temperature^49^. This difference was greater at lower rectal temperatures, hinting towards internal biological temperature drop compensation mechanisms (e.g., brown fat activation^50^ and modulation of peripheral *vs*. core blood flow when body temperature is reduced^51^). Variations in apparent temperature due to differences in concentration of ^13^C-labeled metabolites were determined to be negligible in the range from 5–50 mM (see Fig. 1c, d). For variations on the order of 5–50 mM, this would lead to a maximum deviation of around 4 °C (assuming a chemical shift difference of 12.2 ppm, the apparent respective temperatures are: T_apparent, 5mM_ = 37.6 °C and T_apparent, 50mM_ = 41.5 °C). Importantly, when assuming that the amount of injected pyruvate does not exceed 10% of total blood volume of the animal, concentrations of the metabolite in the bloodstream are expected to be below 8 mM, since pyruvate from the d-DNP device has a concentration of 80 mM. Concentration-dependent differences for animal data should therefore be negligible.

Possible explanations for the apparent temperature drifts (Fig. 5 cf) could be bolus related concentration effects or variations in injected pyruvate temperature, which was not recorded.

Apparent temperature computed from human ^13^C CSI datasets is consistently lower than physiologically reasonable (Fig. 6 and S6) and shows a large variation across the brain (31.6 ± 5.4 °C). Multiple reasons could be the cause for this deviation. Firstly, variations in susceptibility due to tissue microstructure lead to local brain B_0_ changes^52^, which would affect pyruvate and lactate differently, since they are expected to primarily reside in different microstructural compartments with their relative contributions changing dynamically over time. While HP pyruvate is injected intravenously, HP lactate is initially produced intracellularly and is then transported outside of the cell^53–55^. The preclinical data is consistent with this explanation, for which also a large variation across mouse brain apparent temperature maps is observed (37.7 ± 2.8 °C).

Secondly, the uncertainty of the frequency, and therefore temperature, measurement is limited by the accuracy of the spectral fit, which is directly linked to the spectral resolution and noise level. For the four human MRS datasets analyzed in this work, nominal spectral resolution varied between 2 and 20 Hz/pt. To minimize errors from fitting, iterative time-domain fitting algorithms were developed and benchmarked against artificial spectra during this study (see Figure S11) and have been made available for public use on GitHub (https://github.com/Schilling-Lab/pyr-lac-thermometry-study.git). At 3 T, 1 ppm corresponds to 32 Hz for ^13^C nuclei, which then results in a resolution of 48 °C/pt for a spectrum acquired with 20 Hz/pt spectral resolution. Therefore, the fitting algorithm developed in this work is able to achieve sub-resolution accuracy for the resonance frequencies of pyruvate and lactate (see Figure S11d). Lower SNR also leads to a lower accuracy of the fitted temperature, as do worse shims, which was confirmed by simulations (Figure S11b, c). In addition, line broadening of spectra before fitting leads to an error in the fitted frequency and therefore a temperature bias (Figure S11a). Despite those limitations, low SNR spectra acquired at 7 T of thermally polarized pyruvate and lactate in blood at constant temperature (18 °C) and concentration (220 mM) were analyzed and the fitted temperature agrees well with temperature from a Pt100 probe (Figure S11e, f). Thirdly, the concentration of [1-^13^C]pyruvate in clinical d-DNP systems is higher than in the preclinical system used in this work (80 mM vs. 250 mM), which could lead to bolus effects in humans in which concentration-dependent chemical shift difference changes could occur due to slower blood circulation and higher bolus concentration. We tried to investigate this by correlating pyruvate intensity and apparent temperature for healthy human brain data (Figure S5), but found no correlation. Future studies should address this question and investigate the concentration of hyperpolarized pyruvate within a bolus and subsequently in the target tissue explicitly.

Finally, an effect changing the apparent temperature could be due to the fact that previous work has shown that extracellular lactate has a higher frequency than intracellular lactate^56^ by 0.02 ppm, which corresponds to a temperature difference of 1.8 °C (intracellular lactate signal appearing warmer).

For these reasons, this method has limited application for determining accurate absolute temperatures in humans, while it warrants further investigation as an imaging biomarker especially since apparent temperature can be easily retrospectively obtained from hyperpolarized [1-^13^C]pyruvate MRSI datasets. Nevertheless, for spectroscopically-estimated brain temperature from ^13^C MRSI between healthy human brains and patients with GBM, an increased mean brain apparent temperature was found in the GBM case (Fig. 6b). This could also be due to the fact that all patients with GBM were taking high doses of dexamethasone at the time of scanning, which could mildly increase the body temperature^57^, or due to differences in brain microstructure for GBM patients.

Besides an application as a thermometry method, the effects characterized in this work have implications for ^13^C imaging sequences that assume the resonance frequencies to be stable during a scan. For example, in a balanced SSFP sequence as described by Skinner et al.^58^, the relative chemical shift between pyruvate and lactate was assumed to be constant. However, a shift by a few Hz, which could occur for a change in local tissue temperature of a few °C, could lead to a metabolite moving along the response curve, therefore skewing the resulting AUCRs or leading to signal bleeding from one metabolite channel to the other. To avoid this, body and even local organ or tissue temperature needs to be taken into account when using ^13^C MRI sequences that are based on the assumption of constant frequencies of pyruvate and lactate. Additionally, in the setup of sequences for ^13^C MRI, a phantom containing thermally polarized compounds, such as ^13^C lactate, may be used for reference frequency and transmit B_1_ calibrations. If this phantom is placed on the outside of the animal or patient, its temperature will likely be substantially lower than body temperature, and therefore its resonance frequency will also be offset from that in the subject^59^.

To summarize, our method enables temperature or concentration measurements of NMR spectra containing [1-^13^C]pyruvate and [1-^13^C]lactate. Given that both of the metabolites exhibit a temperature and concentration dependent chemical shift, they can also be used independently if another reference molecule, such as [^13^C]urea, is present at the same location in vivo and calibration functions for that molecule have been established, which was outside the scope of this work. Especially for quality control of novel hyperpolarization devices and translation into an automated workflow for clinical use or in vitro studies, this technique would be highly beneficial, since only one NMR spectrum is needed to measure temperature or concentration, assuming the other is known independently. Furthermore, since the temperature information is gained essentially for free with any hyperpolarized pyruvate injection and following spectroscopic sequence, one could investigate changes in apparent temperature due to tumor treatment via chemo- or radiotherapy, with the mentioned limitations to studies.

## Methods

### MR hardware

Preclinical measurements and calibrations were performed on a 7 T preclinical MRI scanner (Discovery MR901 magnet and gradient system, Agilent, Santa Clara, CA, USA; AVANCE III HD electronics, Bruker, Billerica, MA, USA). For mouse abdominal imaging, a 31 mm ^1^H/^13^C dual-tuned volume resonator (RAPID Biomedical, Rimpar, Germany) was used. Mouse brain imaging was conducted using a cryogenically cooled (temperatures: coil 30 K, preamplifier 77 K) linear receive-transmit 20 mm surface coil (Bruker). Phantom measurements at 7 T were carried out in a 10 mm ^13^C solenoid coil (RAPID Biomedical). In vitro calibration measurements at 11.7 T were done on a 500 MHz NMR spectrometer (Bruker). Human brain MRI and MRS was performed on 3 T MR systems (MR750 or MR750w, GE Healthcare, Waukesha WI, USA), using a dual-tuned ^1^H/^13^C quadrature head coil (RAPID Biomedical), as described in the original work^30,43^, a custom built dual-frequency ^1^H/^13^C quadrature head coil (Clinical MR Solutions, LLC, Brookfield WI, USA)^44^ or a 8-channel ^13^C receive coil (Rapid Biomedical) paired with a ^13^C clamshell coil for transmission^45^.

### Hyperpolarization

For preclinical abdominal and in vitro MRI, 26–35 mg 14 M [1-^13^C]pyruvate (Merck, Darmstadt, Germany) or 50–85.7 mg 3 M [1-^13^C]lactate (Merck, Darmstadt, Germany) were polarized together^60^ with 15 mM OX063 trityl radical (GE Healthcare) and 1 mM gadoteric acid (Dotarem, Guerbet, France) in a commercial d-DNP polarizer (Hypersense, Oxford Instruments, Oxford, United Kingdom) at 3.35 T / 1.2 K / 94.139 GHz for more than 45 min. The compound was then rapidly dissolved in 2.9–5.0 ml hot buffered saline solution (80 mM Tris, 80 mM NaOH, 0.1 g/l Na_2_-EDTA) to neutralize pH and reach an average concentration of 80 mM for the ^13^C compound.

For mouse brain MRI, a PHIP-sidearm hydrogenation (SAH)-specific precursor of [1-^13^C]pyruvate was polarized and dissolved in 0.8 ml buffered aqueous solution (16 mM phosphate buffer) using an alpha device of the preclinical polarizer POLARIS (NVision, Germany), yielding a final concentration of 75 mM.

For human MRI, [1-^13^C]pyruvate was polarized in a clinical d-DNP polarizer (SPINlab, 5 T, GE Healthcare), as described in the original work^30,43–45^.

### Temperature reference measurements

Animal and phantom temperature was monitored and recorded using a rectal MR-compatible Pt100 probe (SA Instruments, Stony Brook, NY, USA) with a resolution of ±0.1 °C. For 11.7 T NMR measurements, temperature was set through the NMR software and kept for 5 min to equilibrate before starting of the data acquisition.

### In vitro MRS

Thermal in vitro measurements at 11.7 T used a ^13^C-decoupled sequence (TR = 90 s, FA = 90°, spectral resolution = 1 Hz/pt, averages = 16 (5–50 mM) or 8 (100–600 mM)). Hyperpolarized in vitro calibration measurements at 7 T used a non-selective excitation sequence (FA = 10°, TR = 1.5 s, spectral resolution = 0.5 Hz/pt).

### Thermal phantoms

An aqueous solution containing ^13^C-labeled molecules at a concentration of 600 mM each was prepared by dissolving 62.9 mg of good manufacturing practice (GMP) grade [^13^C]urea in 1200 µl H_2_O. To this solution, 226.7 mg 50% w/w [1-^13^C]Na-lactate and 73 µl 14 M [1-^13^C]pyruvic acid were added (pH 2.3). Due to the low pH, some of the pyruvic acid was converted into parapyruvate, which is also visible in the spectra^61^. Then, the pH was adjusted to 7.88 by titration with NaOH or HCl solution (final volume of 1625 µl). From this solution, seven samples of decreasing concentrations of ^13^C molecules (400, 200, 100, 50, 20, 10, 5 mM) were obtained through serial dilution with pure water (pH = 7.73 ± 0.35, exact values for concentrations and pH given in Table S1). 600 µl of ^13^C-labeled solution was mixed with 60 µl of D_2_O for spectrometer shimming and lock adjustments and filled into 5 mm NMR tubes.

Samples for experiments with varying blood oxygenation were prepared by mixing 38.9 mg [1-^13^C]Na-pyruvate powder (Merck, KGaA, Darmstadt, Germany), 33 µl 9 M [^13^C]urea solution and 76 µl ^13^C 55% w/w Na-lactate solution with 1.5 ml of human blood (drawn into lithium-heparin syringes to prevent blood clotting and be compatible with blood gas analyzers) for a final concentration of approximately 200 mM for each of the metabolites (see Supplemental Information section 4). [1-^13^C]Na-pyruvate was chosen to avoid strongly acidic conditions, which would lead to blood clotting and formation of parapyruvate^61^.

### Hyperpolarized phantoms

For hyperpolarized calibration experiments at different temperatures 152 µl HP [1-^13^C]pyruvate was injected into a 10 mm glass NMR tube, preheated in a water bath and containing 1572 µl fresh PBS buffer (pH 7.35) + 97 µl nicotinamide adenine dinucleotide hydrate (NADH, 250 mM) + 3-9 µl lactate dehydrogenase (LDH) solution (rabbit muscle, Roche, Basel, Switzerland), to reflect in vivo conditions (8 mM, pH = 8.58 ± 0.20, *n* = 8). The amount of LDH was increased at lower temperatures to generate sufficient lactate signal.

To mimic in vivo conditions, 136 µl HP [1-^13^C]pyruvate and [1-^13^C]lactate (in TRIS buffer as described in hyperpolarization section) were injected into a preheated 10 mm glass NMR tube filled with 1.5 ± 0.2 ml of rodent blood, which was prepared in 2 ml blood collection tubes containing ethylenediaminetetraacetic acid (EDTA) to prevent clotting (pH = 7.0 ± 0.2, *n* = 5). Rodent blood gas levels were not controlled.

For both hyperpolarized experiments, NMR acquisitions were started at time of dissolution. Bore temperature was kept stable during the acquisition using a PET Dryer Model B-8 (XPower, City of Industry, CA, USA). See Table S2 for pH and temperature values. After pipetting the HP solutions into the NMR tube close to the bore, a PT-100 temperature sensor was inserted and continuously recorded sample temperature during the acquisition.

### In vivo animal MRSI

12 healthy mice (C57BL/6 mixed with 129S background, age = 144 ± 8 days, weight = 31 ± 4 g, 7 female, 5 male) were anesthetized with 2% isoflurane by volume in 2 l/min 100% O_2_ and manually injected twice with 300 μl 80 mM HP PA via a tail vein catheter. For a previously reported total blood volume for mice of 72 ml/kg^62^, this leads to an upper limit of the pyruvate concentration on the order of 10 mM (10.8 ± 1.4 mM, *n* = 10), which is in line with previously reported values^63^. Apparent temperatures estimated from ^13^C spectra were computed using the linear calibration function for the relative frequency difference of [1-^13^C]pyruvate and [1-^13^C]lactate at 5 mM concentration, which was chosen by assuming that metabolites occur in a lower concentration in perfused organs compared to blood. The animals were divided into two cohorts differing with respect to their rectal temperature (T_rec_) at the second injection. First, animals were kept at around T_rec_ = 38 °C, while for the second injection the temperature was decreased by turning down the bore heating over the course of 1 h, resulting in a T_rec_ = 34–35 °C (first cohort, *n* = 6) or 31–32 °C (second cohort, *n* = 6) before being sacrificed by cervical dislocation (under anesthesia 5% isoflurane, 2 l/min 100% O_2_). All experiments were conducted in accordance with a valid animal license (ROB 55.2-2532.Vet_02-18-91, ROB-55.2-2532.Vet_02-23-70).

Eight animals (four per cohort) underwent a single-slice static 2D free induction decay chemical shift imaging sequence (FID-CSI) placed over the kidneys, starting 17 s post start of the injection, as this is where lactate signal peaking in the kidneys was expected (TR/TE = 235/1 ms, acquisition matrix = 14 × 12, voxel size = 2.2 × 2.2 × 6 mm^3^, FA = 12°, spectral bandwidth = 40 ppm, spectral resolution = 2.2 Hz/pt, acquisition points = 700). One animal per cohort received a dynamic broad excitation bandwidth slice spectroscopy scan to determine differences in metabolism at varying body temperature and time dependent temperature following an injection with HP PA (FA = 10°, TR = 2 s, spectral resolution = 0.55 Hz/pt, acquisition points = 2048). One animal per cohort was scanned using a spectrally-selective 3D balanced steady-state free precession (3D-bSSFP) sequence^39,58^, see Supplemental Information Figures S1, S2.

Additionally, two healthy (C57BL/6, age = 142 ± 4 days, weight = 19.9 ± 0.1 g, both female) mice were imaged using a static 2D FID-CSI (center-out k-space sampling, TR/TE = (88.5, 88.5, 100.4, 174.2/ 1.3) ms, acquisition matrix = (40 × 18, 33 × 18, 35 × 18, 30 × 24) voxel size = (1 x 1 x 3, 1 x 1 x 3, 1 x 1 x 3, 1.33 × 1.33 × 3) mm^3^, FA = (6, 5, 7, 7)°, spectral bandwidth = (40, 40, 70.5, 40) ppm, spectral resolution = (11.8, 11.8, 10.3, 5.9) Hz/pt, acquisition points = 256, 256, 512, 512). CSI slices were positioned horizontally through the mouse brain.

### In vivo human MRSI

Data from four published studies on healthy human brain^43,44^, glioblastoma (GBM)^30^ and kidney metabolism^45^ was re-analyzed and temperature values computed using calibration functions for 5 mM ^13^C metabolites, since an upper limit of the pyruvate concentration in blood could be computed to be on the order of 2 mM (total blood volume of healthy adult humans computed using the Nadler and Lemmens-Bernstein-Brodsky equations^64^) and 5 mM was the lowest calibration function obtained in in vitro characterization experiments. Additionally, at low concentrations the chemical shift difference of PA and LA related to concentration is negligible compared to the shift due to temperature. GBM and renal patients underwent a dynamic broad excitation bandwidth slice spectroscopy scan interleaved with an IDEAL spiral CSI acquisition (the latter being not used for this study)^65^ after intravenous injection with 0.4 ml/kg of 250 mM hyperpolarized [1-^13^C]pyruvate (GBM: TR = 500 ms, FA = 15°, slices = 3, slice thickness = 30 mm, receive bandwidth = 62.5 kHz, acquisition points = 4095, spectral resolution = 15 Hz/point, renal: TR = 500 ms, FA = 15°, slices = 5, slice thickness = 3 cm, Rx bandwidth = 62.5 kHz, acquisition points = 3008, spectral resolution = 20 Hz/point), while healthy volunteers in study 1^43^ were scanned using a static 2D chemical shift imaging sequence (slices = 5, slice thickness = 20 mm, Rx bandwidth = 5 kHz, acquisition points = 256, spectral resolution = 20 Hz/point, matrix size = 10 × 10 with corners of k-space omitted^66^, voxel size = 2×2 cm^2^,) and in study 2^44^ using a slice spectroscopy sequence (TR = 3 s, FA = 6.5°, slices = 4, slice thickness = 15 mm, Rx bandwidth = 5 kHz, acquisition points = 2048, spectral resolution = 2.4 Hz/point). Ethical approvals are found in the respective publications.

### Data analysis

The data was analyzed using Python 3.10. The spectroscopic data was fit in time-domain by iteratively minimizing the sum-of-squares of the difference of the measured data and the modeled free induction decay (FID) data. ChatGPT (OpenAI, San Francisco, CA, USA) was used in assisting with writing analysis code.

Linear fits to in vitro data provided calibration functions for lactate-pyruvate frequency difference to temperature. Calibration curves for 5 mM concentration were applied to compute temperature values from metabolite peak frequencies, which is referred to as apparent temperature in this work due to the other influences on the metric that are described here. For purposes of thresholding, single voxel or repetition temperature uncertainties were calculated from the metabolite peak frequency fit uncertainties via equation S1. For mouse kidney CSI data, resulting apparent temperature maps from fits were 2x interpolated and masked by thresholding pixels with a fit uncertainty below 0.5 °C and an ROI drawn around the animal’s abdomen on the axial T_2_w slice corresponding to the CSI slice. For mouse slice selective spectroscopy data, spectra with pyruvate and lactate peaks above noise level were selected and then spectra with a fit uncertainty above 2 °C excluded. This larger threshold was chosen to account for the difference in SNR between the CSI data and slice-selective spectroscopy data, with CSI spectra having higher SNR due to increased flip angle and signal averaging due to inherent repeated excitations. Mouse brain CSI data was similarly thresholded to pixels with a fit uncertainty below 0.5 °C. For human apparent temperature analysis, values from spectra with a fit uncertainty below 3 °C and peak SNR above 5 were included, due to the lower spectral resolution compared to the animal data. ROI masking was not performed for human brain CSI data.

Uncertainties of averaged subject apparent temperature values were calculated as one standard deviation from the mean of included voxel values or of repeated spectral acquisitions. Uncertainties of mean values for the four different human datasets (healthy brain CSI, healthy brain slice-spectroscopy, healthy kidney and GBM brain) shown in Figure S6 were computed by computing the error of the mean over all subjects in each study. Statistical analysis comparing rectal vs. abdominal temperature from ^13^C spectroscopy was performed using a paired t-test^67^.

It was also found that line broadening changes the resonance frequencies such that the apparent temperature decreases (Figure S11a). Due to this reason, only spectra without line broadening were used in this work.

## Supplementary information

Supplemental_Revision

## Data Availability

The data that support the findings of this study are available from the corresponding author upon reasonable request.

## Code availability

The underlying code for this study in the form of Jupyter Notebooks and a custom Python package are available on GitHub (https://github.com/Schilling-Lab/pyr-lac-thermometry-study.git).
