# Supplementary material for: Temperature dependent chemical shifts of pyruvate and lactate enable in vivo hyperpolarized 13C MRSI thermometry
Source: Npj Imaging. 2025 May 6;3:19. doi: 10.1038/s44303-025-00081-3 (PMC12118740; doi:10.1038/s44303-025-00081-3)
Supplement: Supplementary file 1 — Supplemental_Revision [file 44303_2025_81_MOESM1_ESM.docx]

# Supporting Information

## Spectral fitting methods and fit parameters for concentration and temperature dependent fits

Concentrations of samples used for thermal calibration at 11.7 T shown in Table S1. Temperatures and pH values for calibrations at 7 T are shown in Table S2.

Fit parameters for temperature dependent chemical shifts are shown in Table S3, while concentration dependent chemical shifts are shown in Table S3, to be used in the fit equation S2.

Apparent temperature uncertainty calculation from fitting uncertainty is shown in equation S1.

$dT(f_{pyr-lac},df_{pyr-lac})=|T(f_{pyr-lac}+df_{pyr-lac})$- T($f_{pyr-lac}$)|

**Equation S1: Temperature uncertainty estimation from frequency fit error of relative chemical shift between pyruvate and lactate f_pyr-lac_ and error of that frequency df_pyr-lac_.** Linear calibration function T(f[ppm]) is applied to compute apparent temperature.

| **Pyruvate [mM]** | **Lactate [mM]** | **Urea [mM]** | **pH** |
| --- | --- | --- | --- |
| 615.6 | 616.9 | 633.8 | 7.9 |
| 410.4 | 411.3 | 422.5 | 8.0 |
| 205.2 | 205.6 | 211.3 | 7.9 |
| 100.7 | 100.9 | 103.7 | 8.1 |
| 50.4 | 50.5 | 51.9 | 8.0 |
| 20.1 | 20.2 | 20.7 | 7.7 |
| 10.1 | 10.1 | 10.4 | 7.4 |
| 5.0 | 5.0 | 5.2 | 7.0 |

**Table S1: Concentrations of thermal ^13^C phantoms used for calibration of temperature and concentration dependent chemical shifts of [1-^13^C]pyruvate and lactate.**

| **Sample** | **Temperature [°C]** | **pH** |
| --- | --- | --- |
| Blood 1 | 36.8±0.1 | 6.75 |
| Blood 2 | 29.1±0.1 | 7.27 |
| Blood 3 | 20.7±0.1 | 6.97 |
| Blood 4 | 33.2±0.2 | 7.04 |
| Blood 5 | 35.5±0.1 | 6.95 |
| LDH 1 | 39.6±0.1 | 8.62 |
| LDH 2 | 35.0±0.1 | 8.29 |
| LDH 3 | 30.4±0.2 | 8.27 |
| LDH 4 | 26.0±0.1 | 8.75 |
| LDH 5 | 18.1±0.1 | 8.68 |
| LDH 6 | 35.2±0.2 | 8.55 |
| LDH 7 | 30.2±0.1 | 8.74 |
| LDH 8 | 35.0±0.1 | 8.79 |

**Table S2: Blood and LDH sample temperatures and pH values for measurements at 7 T using hyperpolarized [1-^13^C]pyruvate and [1-^13^C]lactate.**

| **sample row 1** | **LDH** | **Blood** | **5 mM** | **10 mM** | **20 mM** |
| --- | --- | --- | --- | --- | --- |
| **pitch [ppm/°C]** | -0.0144 | -0.0143 | -0.013 | -0.0130 | -0.0131 |
| **crossing [°C]** | 12.7640 | 12.7642 | 12.688 | 12.6961 | 12.7121 |
| **sample row 2** | **50 mM** | **100 mM** | **200 mM** | **400 mM** | **600 mM** |
| **pitch [ppm/°C]** | -0.0134 | -0.0137 | -0.0144 | -0.0154 | -0.0162 |
| **crossing [°C]** | 12.7550 | 12.8150 | 12.9266 | 13.1186 | 13.2889 |

**Table S3: Fit parameters for temperature dependent calibration measurements shown in Figure 1 and 2.**

| **T [°C]** | **A [ppm/mM]** | **B** | **D [ppm]** |
| --- | --- | --- | --- |
| **19.85** | 0.002722 | 0.829179 | 12.419734 |
| **24.85** | 0.002570 | 0.833176 | 12.355617 |
| **29.85** | 0.002420 | 0.837460 | 12.291497 |
| **34.85** | 0.002271 | 0.842064 | 12.227372 |
| **41.85** | 0.002066 | 0.849117 | 12.137591 |

**Table S4: Fit parameters for concentration dependent chemical shift at five temperatures. Data was fitted to a power function as described in equation S1.**

$$f(c)=A \times c^{B}+D$$

**Equation S2: Relationship between frequency (f) and concentration (c) for concentration dependent chemical shift.**

## Dynamic bSSFP acquisition of pyruvate to lactate conversion for two animals at elevated and decreased rectal temperature

One animal per cohort was scanned using a spectrally-selective 3D-balanced steady-state free precession (3D-bSSFP) sequence, as described in previous work[^1,2^](https://www.zotero.org/google-docs/?bkvbZv) (image repetition time=1.9 s, nominal flip angle pyruvate = 5°, nominal FA lactate = 40°, excitation frequency offset pyruvate = -5.07 ppm, excitation frequency offset lactate = 7.11 ppm, RF pulse bandwidth = 818 Hz, assumed frequency difference between metabolites = 920 Hz, spatial resolution = 1.75 mm^3^). Data from these scans is shown in Figure S1.


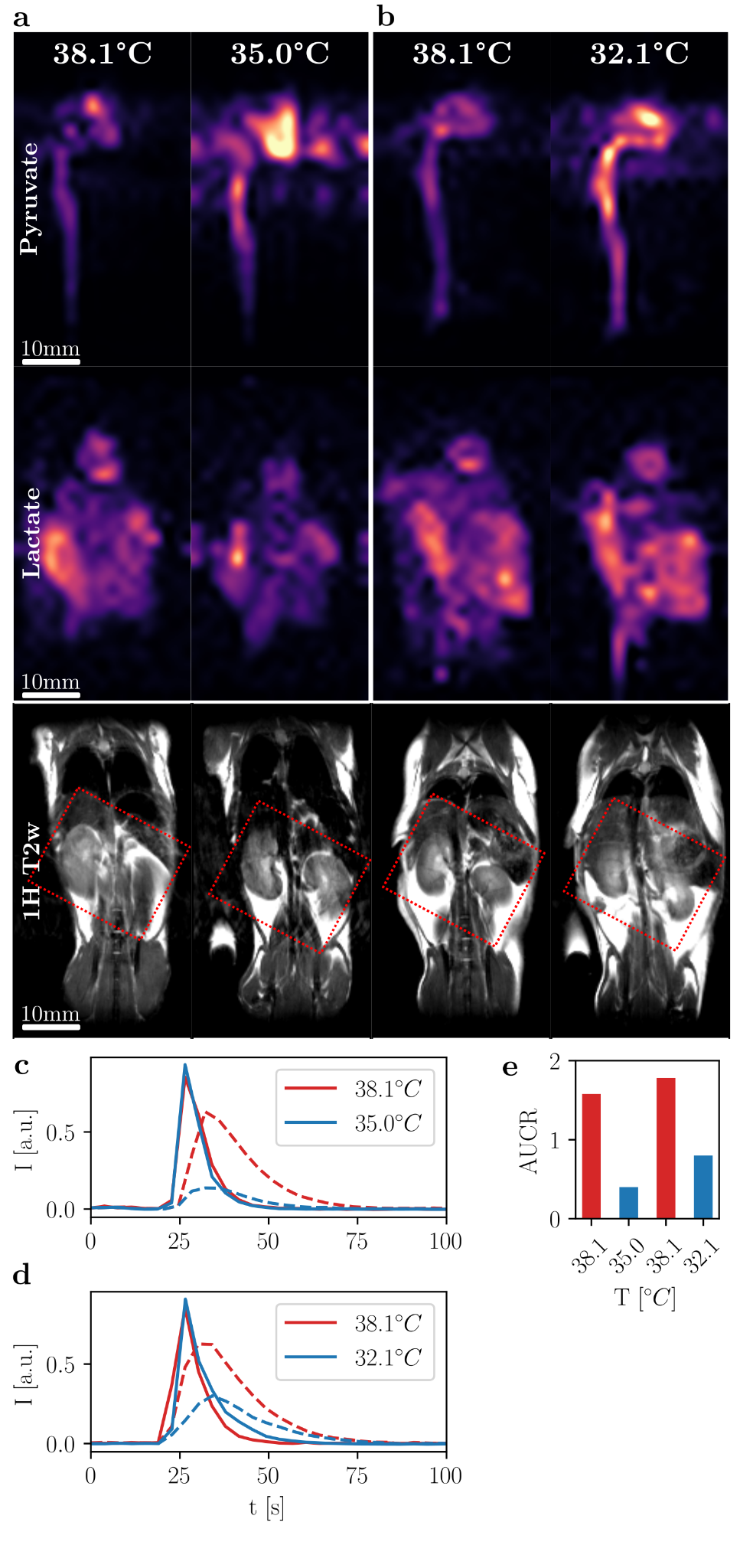


**Figure S1: In vivo 3D-bSSFP imaging of healthy mouse metabolism at different rectal temperatures.** a: Maximum intensity projection signal maps for [1-^13^C]pyruvate and [1-^13^C]lactate for an animal imaged at 38.1 °C and 35.0 °C rectal temperatures as well as anatomical reference images corresponding to the kidney region. b: Data for an animal imaged at 38.1 °C and 32.1°C rectal temperatures. c,d: Pyruvate (solid lines) and lactate (dashed) frequency fit FID amplitude curves of abdomen/kidney ROIs shown in the anatomical images (red dashed) for the two animals depicted in a,b respectively. Curves have been normalized to their respective pyruvate peaks to better visualize the difference in lactate production. e: AUCRs from curves in c,d are shown, with a decrease in AUCR of 75% and 55%, respectively.

### 2.1 Simulations of metabolite positions in bSSFP frequency response profiles

The spectrally-selective bSSFP sequence is based on repeated excitation with low-bandwidth (820 Hz in the described experiments) RF pulses. The repetition time and excitation frequency offset is chosen so that either pyruvate or lactate is placed in a non-zero excitation passband in the resulting frequency response profile, while the other metabolite sits in a passband close to zero intensity[^2^](https://www.zotero.org/google-docs/?I0CpIF). The sequence is sensitive to B_0_ inhomogeneities and additionally to the proper positioning of the metabolites in the response profile. Non-optimal positioning of the response profile with respect to metabolites can lead to longer transient response durations and variability, metabolite contamination from pyruvate into lactate or vice versa and signal loss due to metabolites being positioned in stopbands or areas of passbands with shorter effective T_1_.

To find proper bSSFP sequence parameters, often a [1-^13^C]lactate and a [1-^13^C]pyruvate phantom are used. If this is done at room temperature (17 °C, df_lacpyr_ = 934.6 Hz), with the goal to use the resulting parameters to image in vivo, wrong frequency offsets and repetition times may be chosen (37 °C, df_lacpyr_ = 915.1 Hz).

To set up the bSSFP sequence on a scanner, often a [1-^13^C]lactate phantom is placed next to the subject, and the lactate resonance frequency is taken as a reference to set up the bSSFP frequency parameters. Ignoring frequency differences due to differences in shim, this can still lead to a wrong choice of excitation and receive frequencies due to the difference in temperature between the phantom and the subject. This situation is depicted in Figure S2.

##
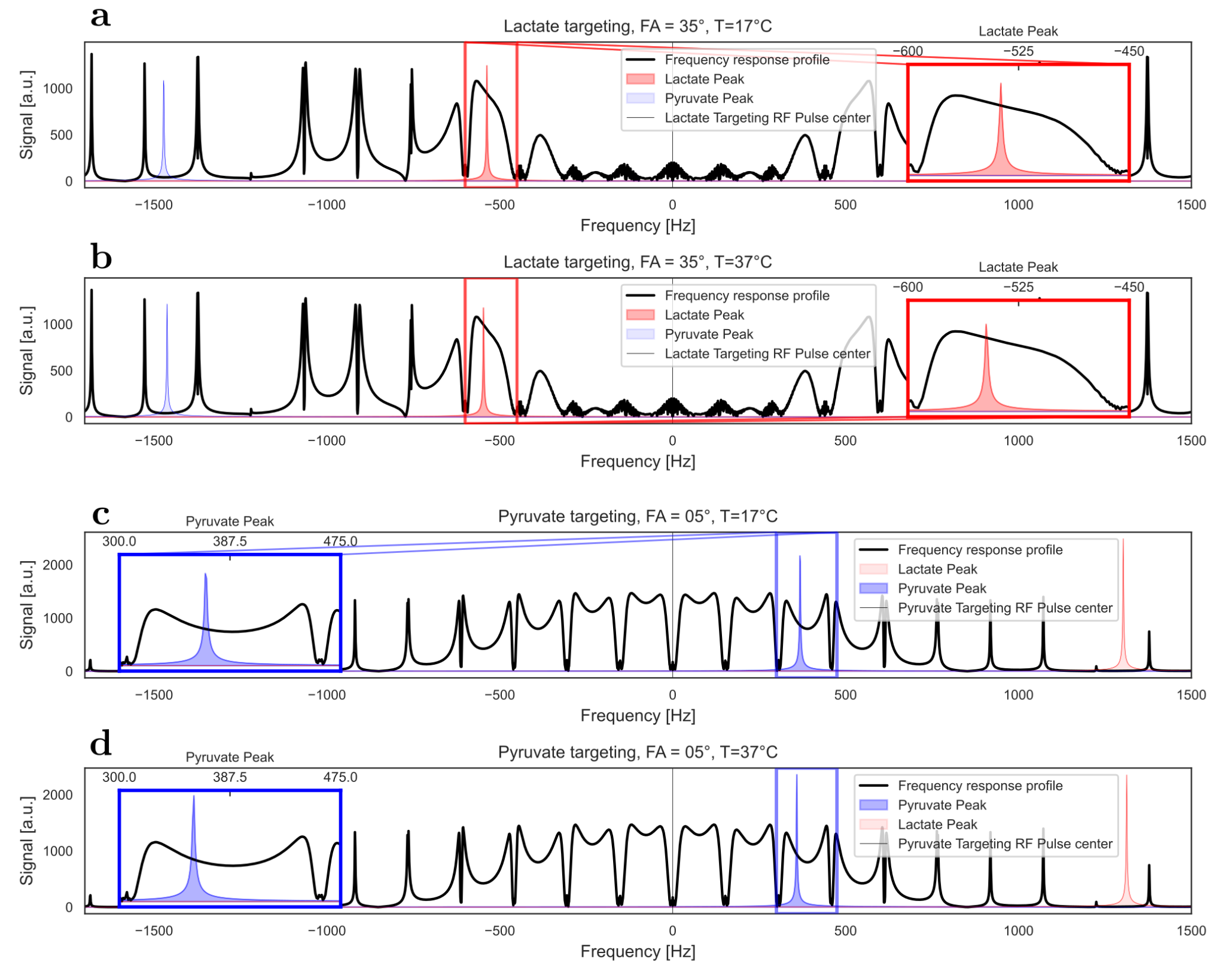


**Figure S2: bSSFP-frequency response profile simulations after repeated excitation with 818 Hz bandwidth RF pulses.** The sequence is assumed to be set up with a lactate phantom at 17 °C (a). a,b: Lactate targeting RF response profiles and positions of lactate and pyruvate peak at 17 °C (a) and 37 °C (b). c,d: Position of metabolites when pyruvate is targeted. It can be seen that both targeted metabolites at 37 °C (b,d) are not well centered in the locally flat and stable part of the passbands, making them more susceptible to B_0_ inhomogeneities.

## Analysis of human brain ^13^C data

Data from an exemplary volunteer published by Kaggie et al[^3^](https://www.zotero.org/google-docs/?DUL7jU) is shown in Figure S3. T_1_w anatomical references, metabolite maps and an apparent temperature map are shown. In Figure S4 apparent temperature maps for all nine volunteers are shown. Values for mean and standard deviation of the apparent temperature are displayed in Table S4. Apparent temperature and area under the curve ratio values for five glioblastoma patients and three slices each, published by Zaccagna et al[^4^](https://www.zotero.org/google-docs/?ldscEt), are displayed in Table S5. Similarly apparent temperature values for four volunteers scanned a total number of seven times and published by Ma et al[^5^](https://www.zotero.org/google-docs/?p6Pgiq) are shown in Table S6. Finally, a summary of all subjects from the four studies is highlighted in Figure S6, corresponding to the box-plots in Figure 6.


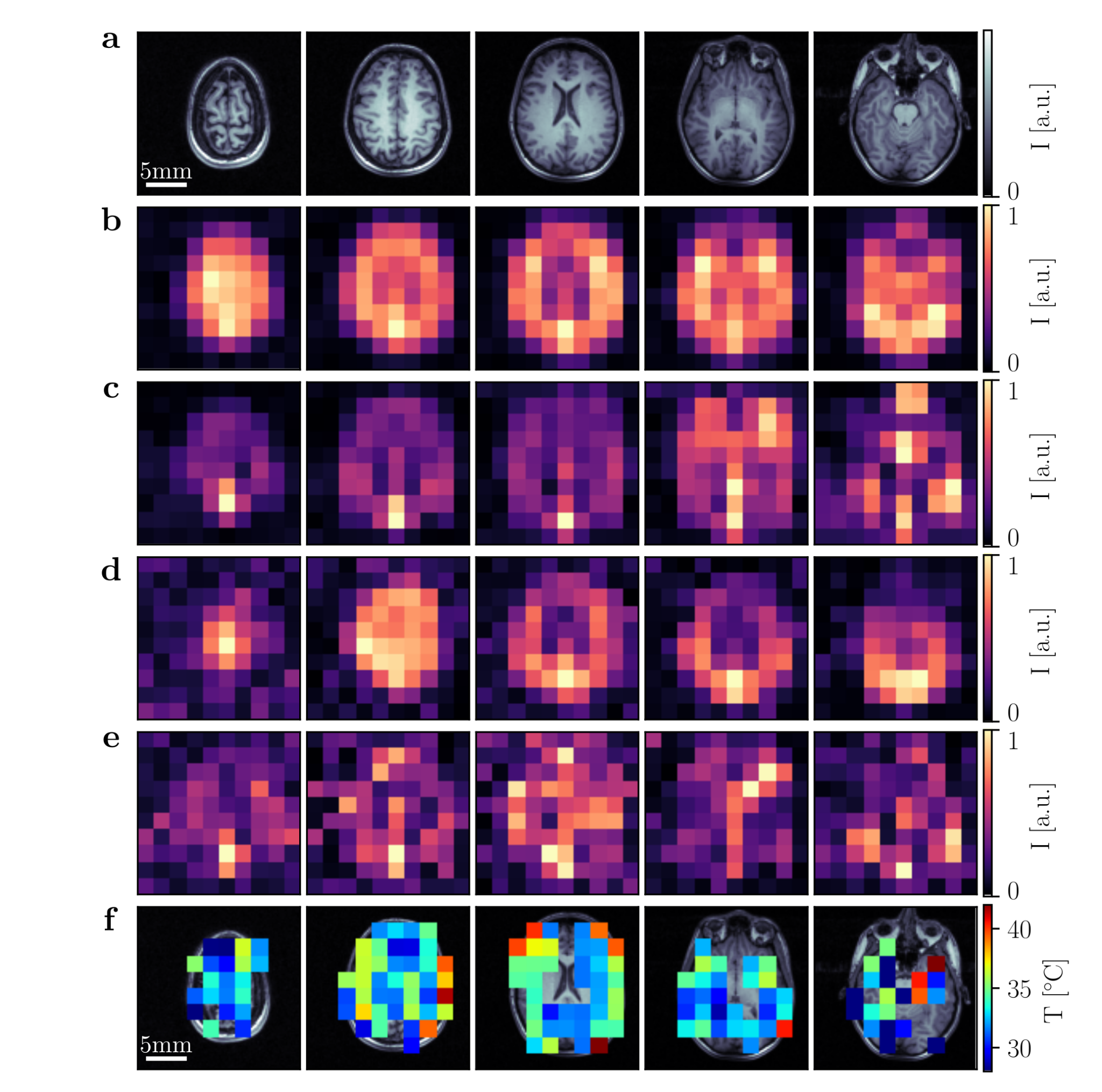


**Figure S3: Healthy human brain MRSI using hyperpolarized [1-^13^C]pyruvate.** Exemplary multi-slice CSI dataset from a healthy volunteer (patient f in Figure S4, slices from left to right). T_1_w anatomical reference slices are shown. Below, lactate, pyruvate, bicarbonate and pyruvate-hydrate intensity maps are plotted for the five slices. Finally, temperature maps computed pyruvate and lactate frequency maps are depicted.
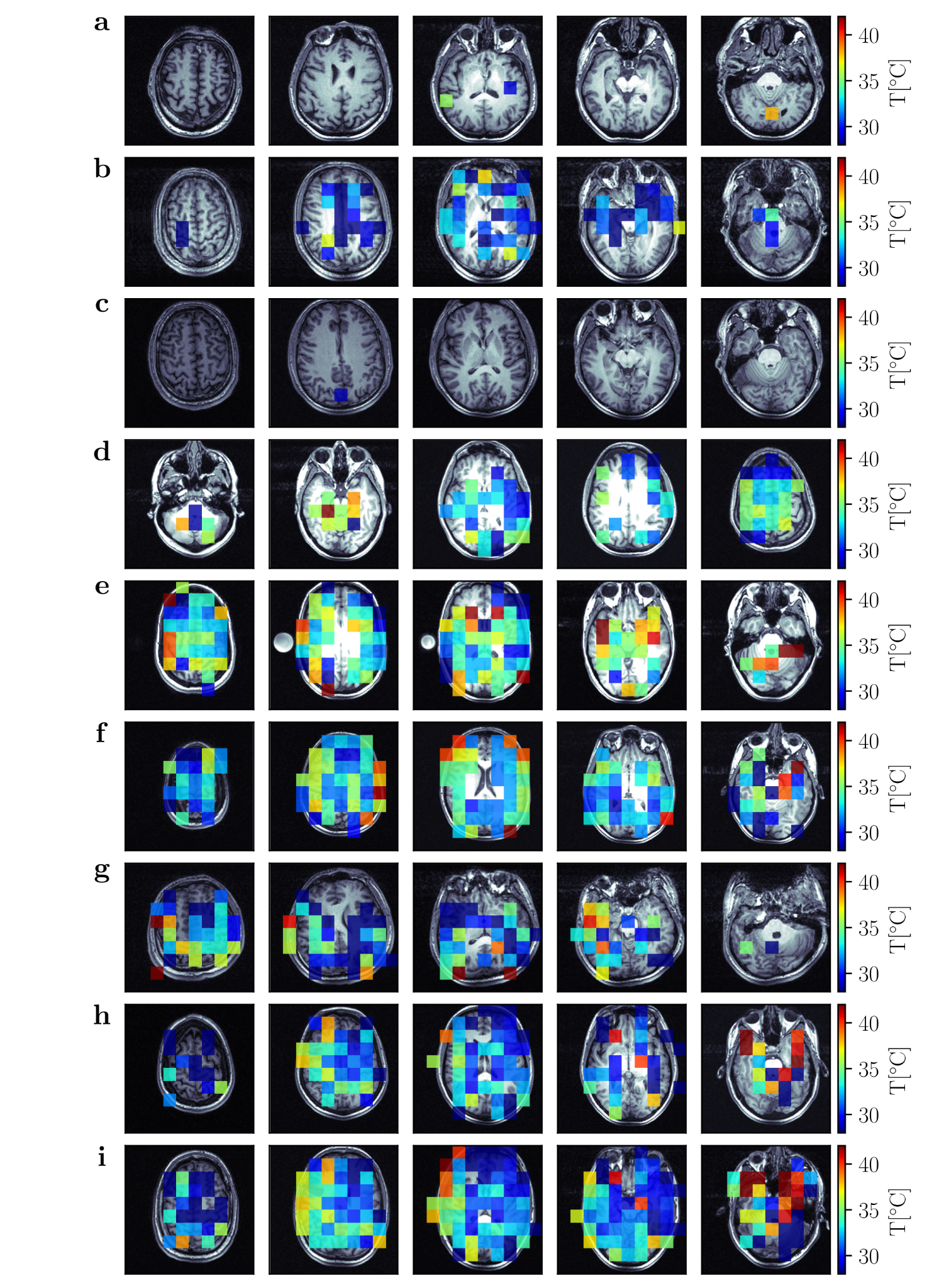


**Figure S4: Healthy human brain temperature maps of nine volunteers (a-i) in five slices (left to right).**


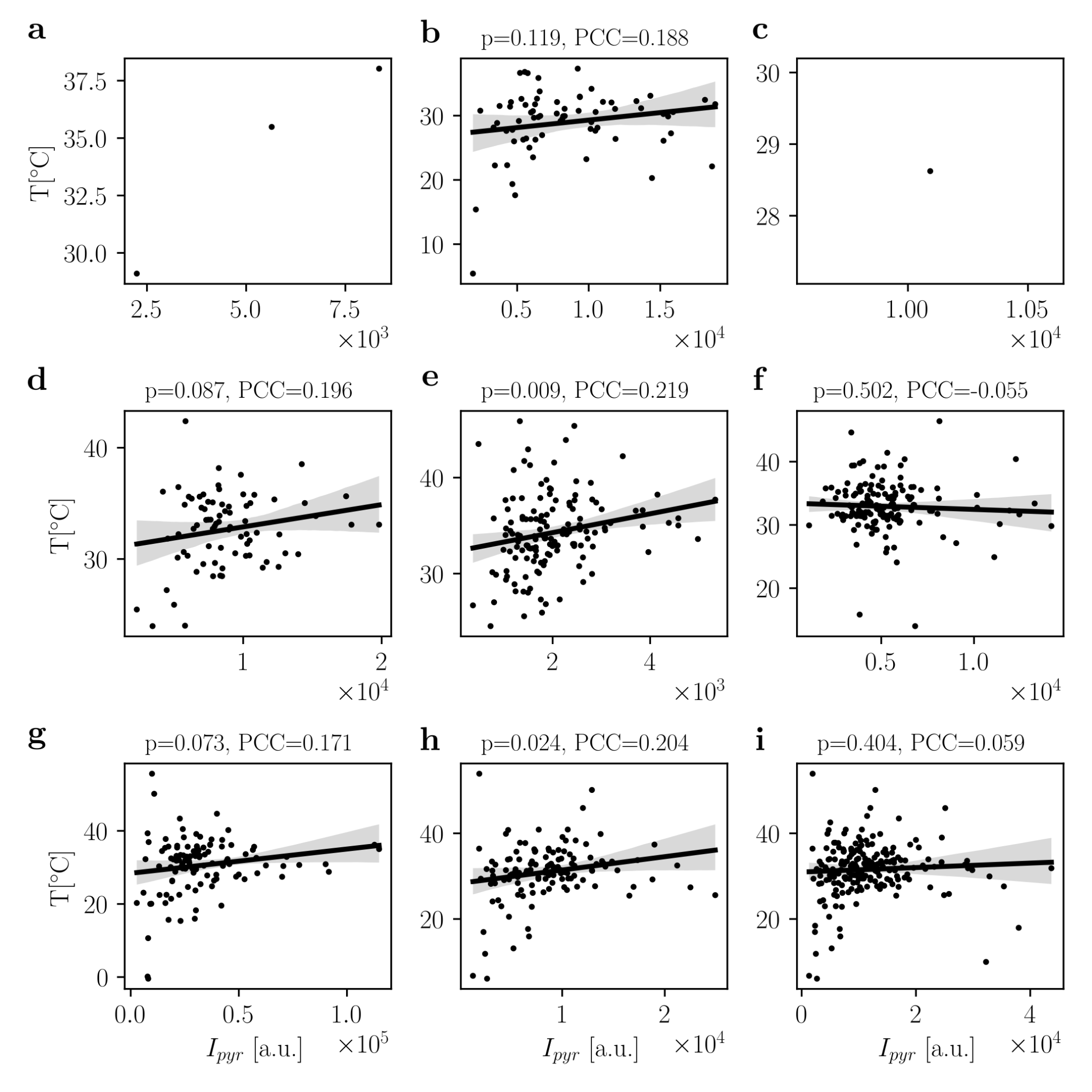


**Figure S5: Correlation of pyruvate signal amplitude and apparent temperature in nine healthy volunteers.** Each panel a-i shows data grouped for all slices, as shown in Figure S4. Panels a and c exhibit not enough datapoints to make any reasonable assumptions about correlation. Linear fits (p-values shown for each panel) and Pearson-correlation coefficients PCC show no significant correlation between pyruvate signal (I_pyr_) and apparent temperature.

| **Patient** | **µ_1_** | **𝜎_1_** | **µ_2_** | **𝜎_2_** | **µ_3_** | **𝜎_3_** | **µ_4_** | **𝜎_4_** | **µ_5_** | **𝜎_5_** | **No. of spectra per datapoint** |
| --- | --- | --- | --- | --- | --- | --- | --- | --- | --- | --- | --- |
| **a** |  |  |  |  | 32.3 | 3.2 |  |  | 38.0 | 0.0 | 0/0/2/0/1 |
| **b** | 21.2 | 0.9 | 28.8 | 3.2 | 30.9 | 3.6 | 26.3 | 7.2 | 31.0 | 1.9 | 2/18/28/18/4 |
| **c** |  |  | 28.6 | 0.0 |  |  |  |  |  |  | 0/1/0/0/0 |
| **d** | 33.0 | 3.9 | 36.0 | 3.8 | 31.9 | 2.6 | 32.1 | 2.5 | 32.5 | 3.2 | 5/9/25/14/24 |
| **e** | 34.1 | 3.3 | 33.6 | 3.8 | 33.4 | 4.0 | 36.3 | 3.3 | 38.4 | 4.3 | 32/36/45/21/7 |
| **f** | 31.7 | 2.7 | 33.7 | 3.0 | 34.2 | 3.0 | 32.7 | 2.2 | 30.6 | 7.4 | 21/41/40/29/21 |
| **g** | 32.3 | 5.9 | 28.6 | 7.6 | 29.7 | 10.4 | 32.7 | 5.4 | 31.5 | 3.5 | 28/32/29/20/2 |
| **h** | 23.6 | 9.2 | 31.9 | 5.4 | 30.7 | 3.9 | 30.8 | 4.5 | 36.4 | 9.5 | 12/31/41/23/15 |
| **i** | 27.3 | 8.0 | 32.4 | 4.9 | 31.3 | 4.8 | 31.6 | 3.6 | 34.0 | 9.7 | 23/43/57/50/31 |

**Table S5: Healthy human brain temperature for nine volunteers in five slices.** Mean (µ) and standard deviation (𝜎) per slice are shown. Values correspond to plots in Figure S4. Slices without sufficient signal are left blank.

|  | **Temperature per slice [°C]** | | | | | | **Area under the curve ratio per slice** | | | | | | **No. of spectra per datapoint** |
| --- | --- | --- | --- | --- | --- | --- | --- | --- | --- | --- | --- | --- | --- |
| **Pat.** | **µ_1_** | **𝜎_1_** | **µ_2_** | **𝜎_2_** | **µ_3_** | **𝜎_3_** | **µ_1_** | **𝜎_1_** | **µ_2_** | **𝜎_2_** | **µ_3_** | **𝜎_3_** |  |
| 1 | 35.8 | 1.0 | 34.0 | 0.8 | 36.6 | 1.8 | 0.4 | <0.01 | 0.4 | <0.01 | 0.2 | <0.01 | 13/13/12 |
| **2** | 35.6 | 0.6 | 34.2 | 0.8 | 35.9 | 0.7 | 0.3 | <0.01 | 0.2 | <0.01 | 0.1 | <0.01 | 12/12/11 |
| **3** | 29.0 | 2.3 | 34.0 | 0.6 | 33.4 | 0.6 | 0.3 | <0.01 | 0.3 | <0.01 | 0.3 | <0.01 | 13/14/13 |
| **4** | 39.0 | 2.5 | 36.2 | 1.3 | 33.7 | 0.9 | 0.4 | <0.01 | 0.3 | <0.01 | 0.2 | <0.01 | 14/17/15 |
| **5** | 37.1 | 0.8 | 36.0 | 1.9 |  |  | 0.3 | <0.02 | 0.2 | <0.01 |  |  | 12/13 |
| **6** | 35.7 | 0.9 | 33.9 | 1.2 | 36.5 | 2.1 | 0.3 | <0.01 | 0.3 | <0.01 | 0.2 | <0.01 | 18/18/18 |

**Table S6: Human glioblastoma data for six patients in three slices**[^4^](https://www.zotero.org/google-docs/?NPPahb)**.** Mean (µ) and uncertainty (𝜎) per slice are shown. Slices without sufficient signal are left blank.

|  | **Temperature per slice [°C]** | | | | | | | | **Injection no.** | **No. of spectra per datapoint** |
| --- | --- | --- | --- | --- | --- | --- | --- | --- | --- | --- |
| **Subject** | **µ_1_** | **𝜎_1_** | **µ_2_** | **𝜎_2_** | **µ_3_** | **𝜎_3_** | **µ_4_** | **𝜎_4_** |  |  |
| 1 | 29.2 | 3.6 | 30.1 | 3.7 | 28.3 | 3.4 | 25.1 | 3.1 | 1 | 74/30/29/55 |
| **2** | 32.9 | 3.0 | 33.1 | 3.8 | 37.5 | 7.0 | 32.4 | 4.3 | 1 | 93/101/78/15 |
| **2** | 39.0 | 2.9 | 28.2 | 4.6 | 34.3 | 5.1 | 31.7 | 5.7 | 2 | 13/42/12/9 |
| **3** | 31.9 | 5.1 | 29.4 | 2.5 | 31.9 | 5.0 | 35.4 | 3.9 | 1 | 23/40/36/24 |
| **3** | 29.2 | 3.6 | 30.1 | 3.7 | 28.3 | 3.4 | 25.1 | 3.1 | 2 | 74/30/29/55 |
| **4** | 32.9 | 3.0 | 33.1 | 3.8 | 37.5 | 7.0 | 32.4 | 4.3 | 1 | 93/101/78/15 |
| **4** | 39.0 | 2.9 | 28.2 | 4.6 | 34.3 | 5.1 | 31.7 | 5.7 | 2 | 13/42/12/9 |

**Table S7: Healthy human slice-selective brain data for four healthy volunteers in four slices**[^5^](https://www.zotero.org/google-docs/?bCSuyf)**.** 8-channel data was analyzed separately for each channel, leading to the high number of spectra. Subjects 2-4 were injected twice with HP PA.

|  |  | **Temperature per slice [°C]** | | | | | | | | | | **Exam no.** | **No. of spectra per datapoint** |
| --- | --- | --- | --- | --- | --- | --- | --- | --- | --- | --- | --- | --- | --- |
| **Dataset** | **Subject** | **µ_1_** | **𝜎_1_** | **µ_2_** | **𝜎_2_** | **µ_3_** | **𝜎_3_** | **µ_4_** | **𝜎_4_** | **µ_5_** | **𝜎_5_** |  |  |
| 1 | 1 | 32.3 | 1.0 | 32.8 | 1.8 | 30.6 | 2.2 | 34.6 | 1.4 |  |  | 1 | 14/54/61/35/0 |
| **2** | **2** | 32.7 | 0.2 | 30.9 | 1.1 | 33.1 | 1.8 |  |  |  |  | 1 | 4/11/8 |
| **3** | **3** |  |  | 29.8 | 1.5 | 31.1 | 1.7 | 32.4 | 1.4 |  |  | 1 | 0/20/54/53/0 |
| **4** | **4** | 35.6 | 1.2 | 33.9 | 1.5 | 32.2 | 0.9 | 31.7 | 1.2 | 33.8 | 0.3 | 1 | 24/26/22/10/4 |
| **5** | **5** |  |  | 30.0 | 0.8 | 33.3 | 0.9 | 31.7 | 1.0 | 31.3 | 3.9 | 1 | 0/9/17/13/4 |
| **6** | **5** | 38.4 | 0.4 | 32.0 | 1.3 | 31.8 | 0.9 | 32.0 | 1.5 |  |  | 2 | 6/22/15/11/0 |
| **7** | **6** | 36.5 | 2.2 | 33.6 | 0.8 | 35.2 | 1.1 | 35.7 | 1.0 | 39.5 | 1.8 | 1 | 40/24/36/40/2 |
| **8** | **7** | 34.4 | 1.8 | 35.7 | 1.8 | 35.9 | 2.1 | 38.2 | 4.5 |  |  | 1 | 23/26/29/3/0 |
| **9** | **8** | 36.4 | 0.7 | 35.2 | 1.2 | 34.3 | 1.7 |  |  |  |  | 1 | 5/9/5/0/0 |
| **10** | **9** | 34.7 | 2.0 | 32.3 | 3.5 |  |  |  |  |  |  | 1 | 7/2/0/0/0 |
| **11** | **12** | 33.1 | 2.1 | 29.0 | 2.5 | 23.8 | 3.1 |  |  |  |  | 1 | 55/23/3/0/0 |

**Table S8: Human slice-selective kidney data for 12 patients in 3-5 slices**[^6^](https://www.zotero.org/google-docs/?Ozcdlw)**.** 8-channel data was analyzed separately for each channel. Subjects 5 and 7 were injected twice with HP PA. Subject 10,11 (exam 1 and 2) and 7 (exam 2), did not have sufficient SNR. Rows without a value below the fit error threshold are left empty.


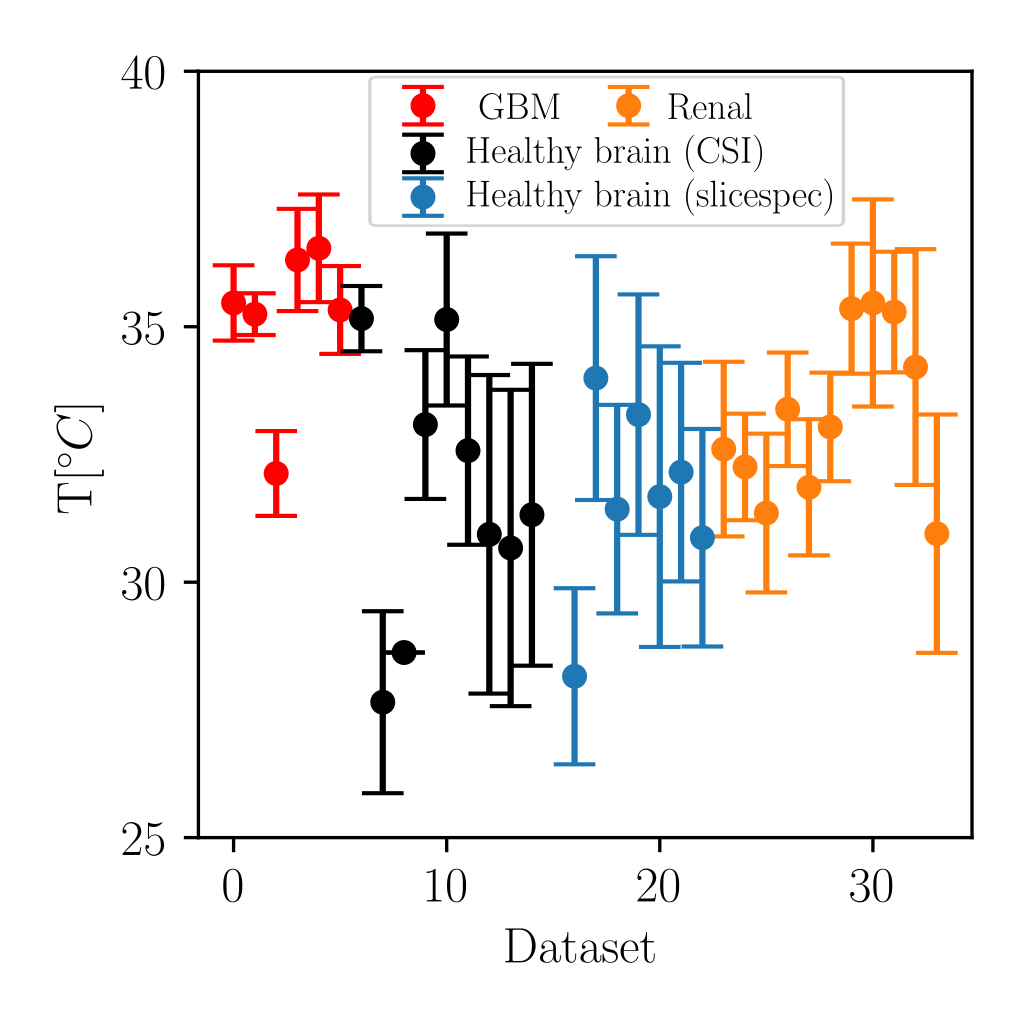


**Figure S6: Human brain temperature from ^13^C MRSI for all datasets.** Mean multi-slice brain temperatures for voxels with fit accuracy better than 3 °C were computed, assuming 5 mM concentration of metabolites. Standard deviations are given as error bars. Healthy CSI[^3^](https://www.zotero.org/google-docs/?etiGIm), Healthy slice-selective spectroscopy[^5^](https://www.zotero.org/google-docs/?2xTqWl) and glioblastoma[^4^](https://www.zotero.org/google-docs/?avRwka) data corresponding to box-plots in Figure 6. Data for all slices and all datasets is shown in Tables S5-8.

## Varying blood oxygenation of human blood containing thermally polarized ^13^C metabolites

To assess how ^13^C labelled pyruvate, lactate and urea resonance frequencies change with changing blood oxygenation, human blood from a healthy volunteer (29 y, male) was drawn into lithium-heparin syringes (Sarstedt AG & Co. KG, Nümbrecht, Germany). 38.9 mg [1-^13^C]Na-pyruvate powder (Merck, KGaA, Darmstadt, Germany) was dissolved in roughly 1 ml of blood using a vortexer. The solution was then pipetted into 10 mm glass NMR tubes. Then, 33 µl of an aqueous 9 M ^13^C research-grade urea solution as well as 76 µl ^13^C 55% w/w Na-lactate solution was added. Finally, the tube was filled to approximately 1.5 ml volume using residual blood and closed using a custom 3D printed plastic plug with three small holes for gas exchange and temperature control. Two samples were prepared for each of the four experiments shown here. The first one was connected to an oxygen gas flask and plastic catheter connected with one hole of the plastic plug, to achieve a highly oxygenation state in the blood. The second one was connected in a similar fashion to a nitrogen gas flask. Gas flow rate in both setups were controlled using standard animal anesthesia devices (set to the lowest possible setting, i.e. 0.2 l/min flow rate). Samples were agitated and turned every five minutes to equally distribute the gas within the vial and blood and avoid clotting as well as drying. In total, agitation samples were placed under constant gas flow at room temperature (18 °C) for 30 minutes. The general scheme used was adapted from[^7^](https://www.zotero.org/google-docs/?Wgd0Og).

MR experiments were conducted at a 7 T preclinical MRI, using a ^13^C solenoid coil as described above. Reference frequency and power adjustment was performed using a 2 M ^13^C lactate, 2 mM DOTA in water phantom before start of the oxygenation/deoxygenation process. Once the two samples had equilibrated for 30 min, temperature was checked in the tubes (T_high,oxy_=18.5±0.4 °C and T_low,oxy_=18.6±0.5 °C, n=4) and then first the highly oxygenated sample was removed from the plastic catheter connected to the oxygen flask and immediately sealed airtight using parafilm. The sample was placed in the solenoid coil and six repetitions of a non-localized spectroscopy sequence (FA=90°, TR=10 s, spectral resolution=1.5 Hz/pt) were acquired. The tube was then removed and the same process repeated with the lower oxygenated sample. Finally, blood from both samples was drawn into standard Li-Heparin syringes (1 ml, Sarstedt) using the same catheters as used for gasflow. Syringes were evacuated and transferred to a clinical blood gas analyzer in another department (RapidPoint 500e, Siemens Healthineers AG, Forchheim, Germany). A standard blood gas analysis protocol was performed by a trained technician. Results for pH, sO_2_, pO_2_, pO_2_ (corrected), pCO_2_, Hct and FMetHb are shown in Table S8.

MR spectra were averaged and then fitted to extract resonance frequencies. Relative frequency change between pyruvate and lactate does not change systematically with oxygenation/deoxygenation (Figure S7a). However values for lower oxygenated blood appear to be spread out more than highly oxygenated blood. Absolute frequency values compared between high oxy./ low oxy. measurements follow for the most part the expected trend of decreasing with deoxygenation (Figure S7b-d) for all three metabolites. One of the experiments conducted (Exp. 3) was performed using a different shim setting on the spectrometer to improve SNR. All other experiments were performed using the same shim setting. A possible reason for these inconsistencies could be that all experiments suffer from changes in local B_0_ due to slight differences in volume between high oxy. / low oxy. samples. Identical volumes (with deviations below ±200 µl) are difficult to obtain due to the oxygenation / deoxygenation process where fluid is lost easily due to turning and soft shaking of the NMR tubes containing the blood.


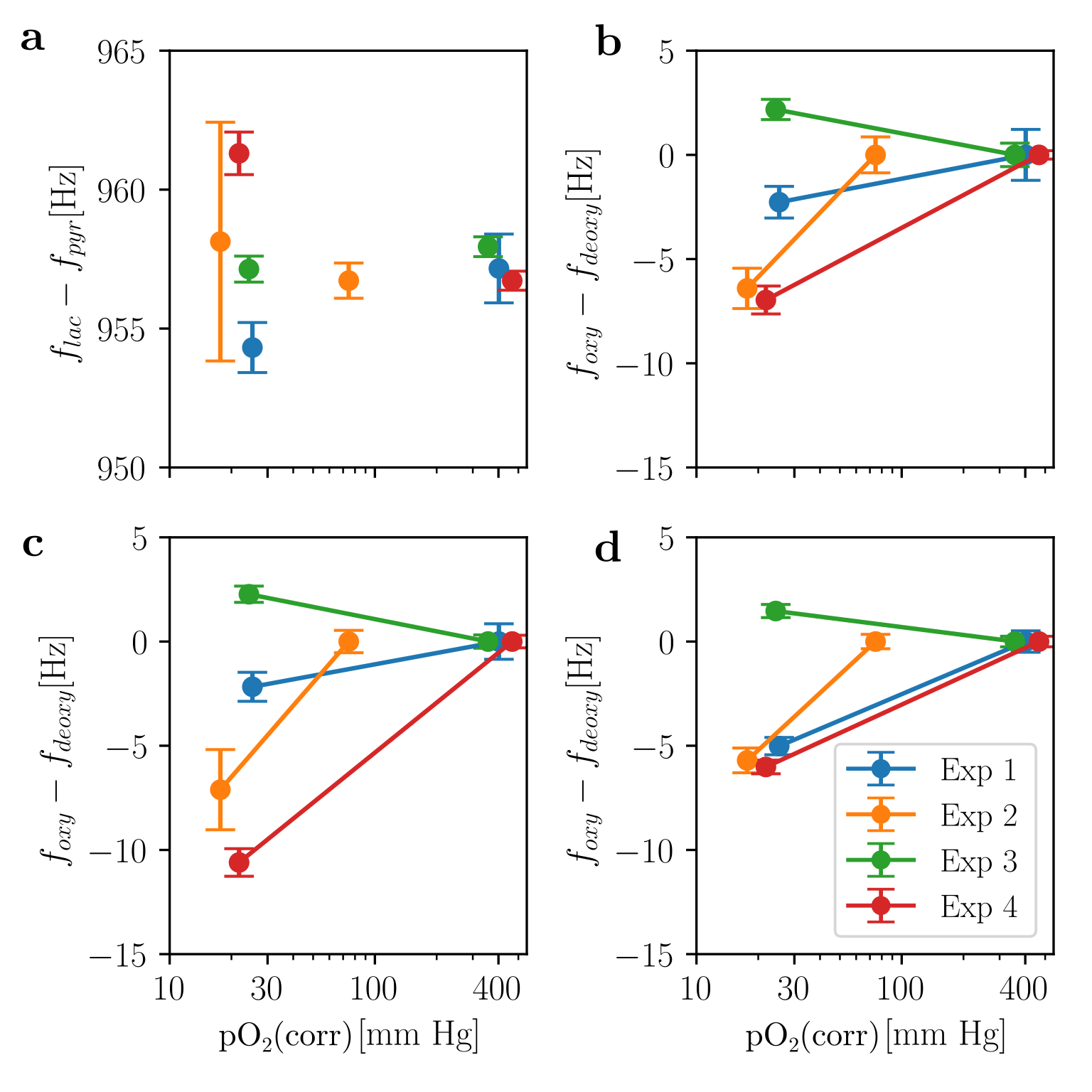


**Figure S7: Varying blood oxygenation for thermally polarized 200 mM ^13^C pyruvate, lactate and urea dissolved in 1.5 ml human blood.** Relative peak distance between pyruvate and lactate (a) does not appear to change with oxygenation. Absolute peak positions of urea (b), pyruvate (c) and lactate (d) move to lower frequencies for three of four experiments when oxygenation is lowered. Experiments 1, 2 and 4 conducted using identical shim settings on the spectrometer. For experiment 3, different shim settings were used.

| **Experiment** | **Gas** | **sO2 (37°C)** | **pO2**  **(37°C)** | **pO2**  **(corr)** | **Hct** | **pH** | **T [°C]** |
| --- | --- | --- | --- | --- | --- | --- | --- |
| 1 | O_2_ | 99.0 | 566.2 | 402.0 | 44 | 7.54 | 18.6 |
| 1 | N_2_ | 74.7 | 35.5 | 25.3 | 48 | 7.53 | 18.8 |
| 2 | O_2_ | 97.3 | 106.4 | 74.6 | 52 | 7.544 | 17.9 |
| 2 | N_2_ | 52.7 | 25.2 | 17.6 | 46 | 7.576 | 17.9 |
| 3 | O_2_ | 98.8 | 502.3 | 141.4 | 44 | 7.592 | 18.5 |
| 3 | N_2_ | 75.2 | 34.4 | 20.0 | 44 | 7.614 | 18.4 |
| 4 | O_2_ | 99.2 | 651.2 | 466.7 | 41 | 7.58 | 19.1 |
| 4 | N_2_ | 73.6 | 30.4 | 21.8 | 47 | 7.613 | 19.1 |

**Table S9: Clinical blood gas analysis measurements for four experiments.** Gas analysis was conducted at 37 °C by default in the device. pO_2_ (corr) was corrected for measurement temperatures using calibration functions found in [^8,9^](https://www.zotero.org/google-docs/?6gB85F).

## Influence of pH-induced changes in chemical shifts

**Figure S8: Influence pH on apparent temperature measurements.** a: Spectra from an NMR titration series for [1-^13^C]pyruvate. At highly basic pH (topmost spectra, base-mediated formation of pyruvate multimers is observable. b: Spectra from an NMR titration series for [1-^13^C]lactate. c: Chemical shift of [1-^13^C]pyruvate as a function of pH and fit curve using the Hendersson-Hasselbalch equation. The inset shows a magnification of the in vivo relevant pH region. Chemical shift readings at three different pH values are determined from the fit curve and stated with three digits precision. d: Chemical shift of [1-^13^C]lactate as a function of pH and fit curve using the Hendersson-Hasselbalch equation. The inset shows a magnification of the in vivo relevant pH region. Chemical shift readings at three different pH values are determined from the fit curve and stated with three digits precision.
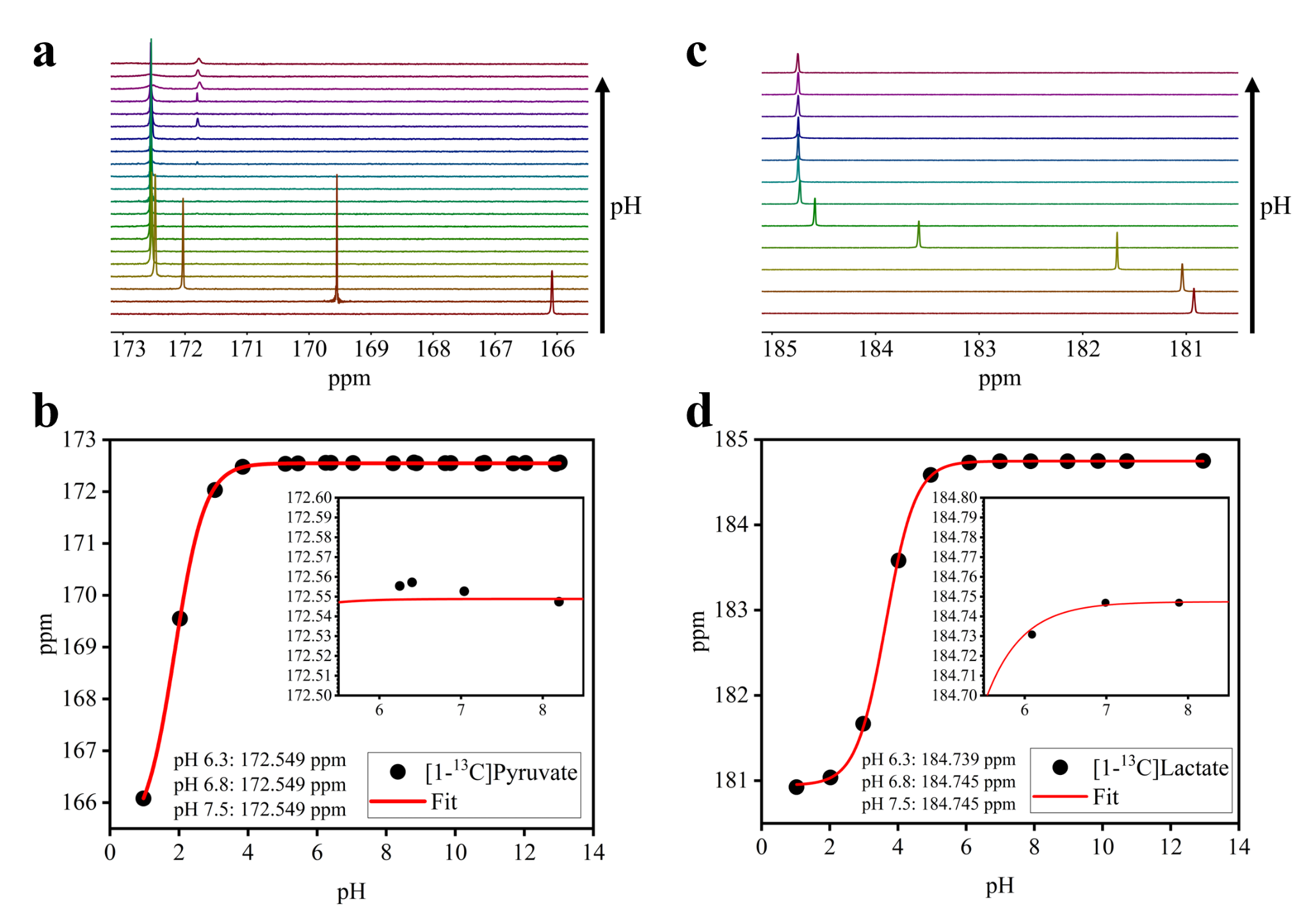


To assess the influence of pH on the accuracy of the temperature measurement using [1-^13^C]pyruvate and [1-^13^C]lactate, NMR titration series being acquired in previous studies (Hundshammer and Grashei 2019 (<https://doi.org/10.1002/cphc.201801098>) and Grashei 2021 (<https://doi.org/10.3390/ph14040327>)) were analyzed. In short, stock solutions containing either 250 mM [1-^13^C]pyruvate or [1-^13^C]lactate in water containing 10% D_2_O were titrated to different pH values using 10 M KOH or 37% HCl. For each pH step, pH values were measured using a conventional pH electrode (N 6000 A electrode on a ProLab 4000 multiparameter benchtop meter, SI analytics, Mainz, Germany) before and after NMR acquisition to assure pH stability. Spectra were acquired on a 14.1 T Bruker AV 600 NMR spectrometer (Bruker Biospin, Billerica, USA) using a pulse-and-acquire experiment with inverse-gated decoupling and flip angle 30°, repetition time 2s, 30 kHz spectral width, 30192 points, 32 averages. Spectra were analyzed using MestReNova 15.0.1 (Mestrelab, Santiago de Compostela, Spain). Prior to fitting, spectra were phased manually and baseline-corrected. No line broadening was applied. Chemical shifts were determined from line fitting and chemical shift values as a function of pH were fitted in OriginPro 2018b (OriginLab, Northampton, USA) using the Hendersson-Hasselbalch equation. From the NMR titration series both [1-^13^C]pyruvate (Figure S8a) and [1-^13^C]lactate (Figure S8b) show a strong pH dependence of their ^13^C chemical shift. However these regions are located close to the p*K*_a_-values (pyruvate: 2.49, lactate: 3.90) of both molecules. For in vivo applications, the relevant pH range is approximately pH 6.3 - 7.5 (Grashei 2023 (https://doi.org/10.1038/s41467-023-40747-3)). The fitting of the chemical shifts as a function of pH indicates no relevant change in NMR frequency (< 0.001 ppm) for [1-^13^C]pyruvate within this pH range (Figure 8c). As [1-^13^C]lactate has a more basic acid dissociation constant, its chemical shift as a function of pH (Figure 8d) reveals that pH-induced NMR frequency changes start to occur below pH 6.5 (Figure 8d inset). Across the in vivo relevant range, this results in a maximum change of 0.006 ppm. Taking together the pH effects on pyruvate and on lactate and comparing it to the thermometry calibration in Table 1 in the manuscript, the pH-related uncertainty of the temperature measurement is < 0.5°C, thereby indicating pH to be of negligible influence.

## Healthy mouse brain MRSI using HP pyruvate

During a different study the brains of two healthy mice were scanned using parahydrogen-polarized [1-^13^C]pyruvate and a cryogenically cooled surface coil. Data was analyzed using the same calibration functions and methods as for human data and abdominal rodent data shown. Brain apparent temperature maps were computed and are shown in Figure S9.


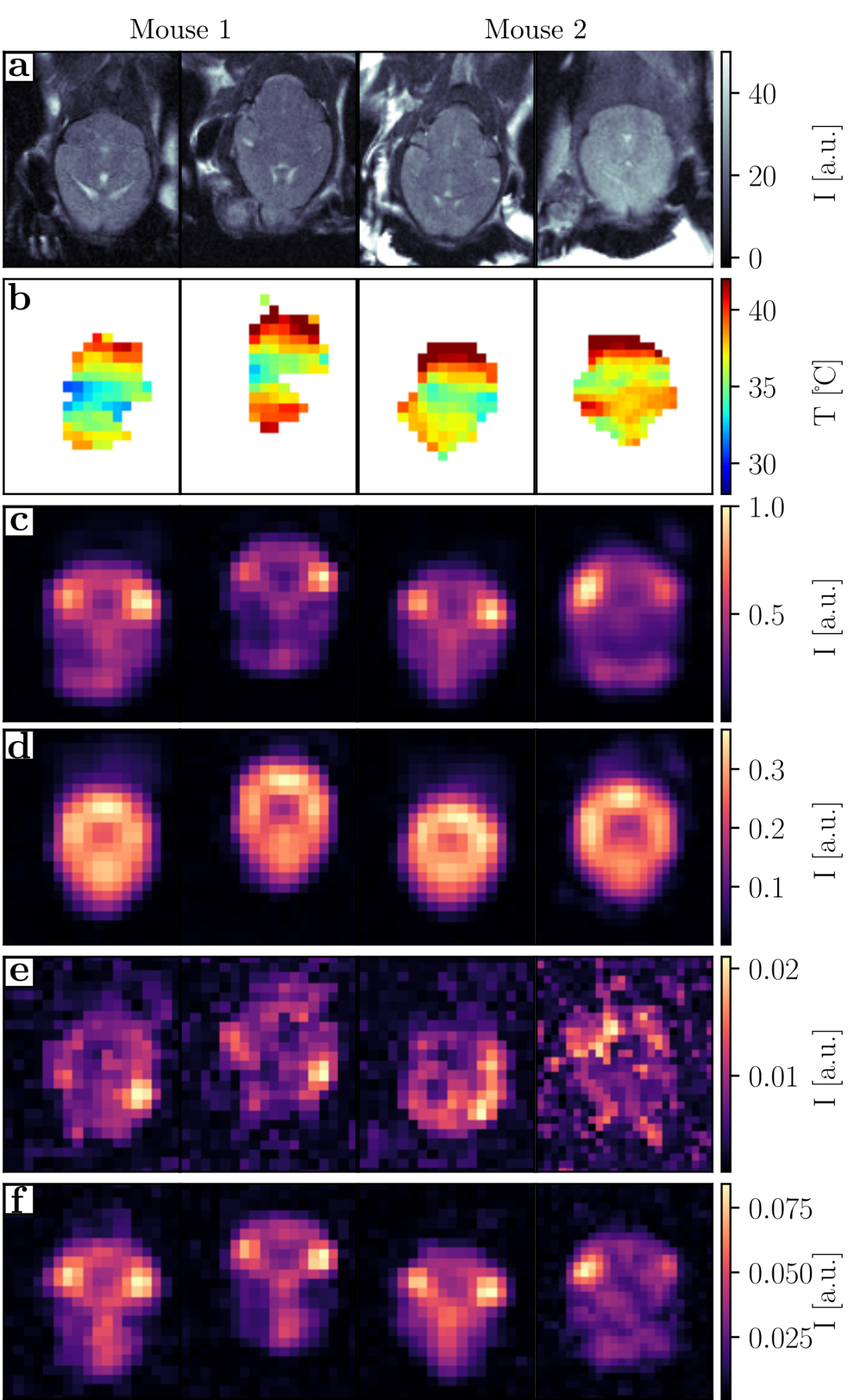


**Figure S9: Healthy mouse brain metabolism and apparent temperature maps in two mice, scanned twice each.** a: T_1_w proton references. b: apparent temperature maps. c: pyruvate, d: lactate, e: bicarbonate and f: pyruvate hydrate amplitude maps. Histograms of apparent temperature maps are shown in Figure S10.


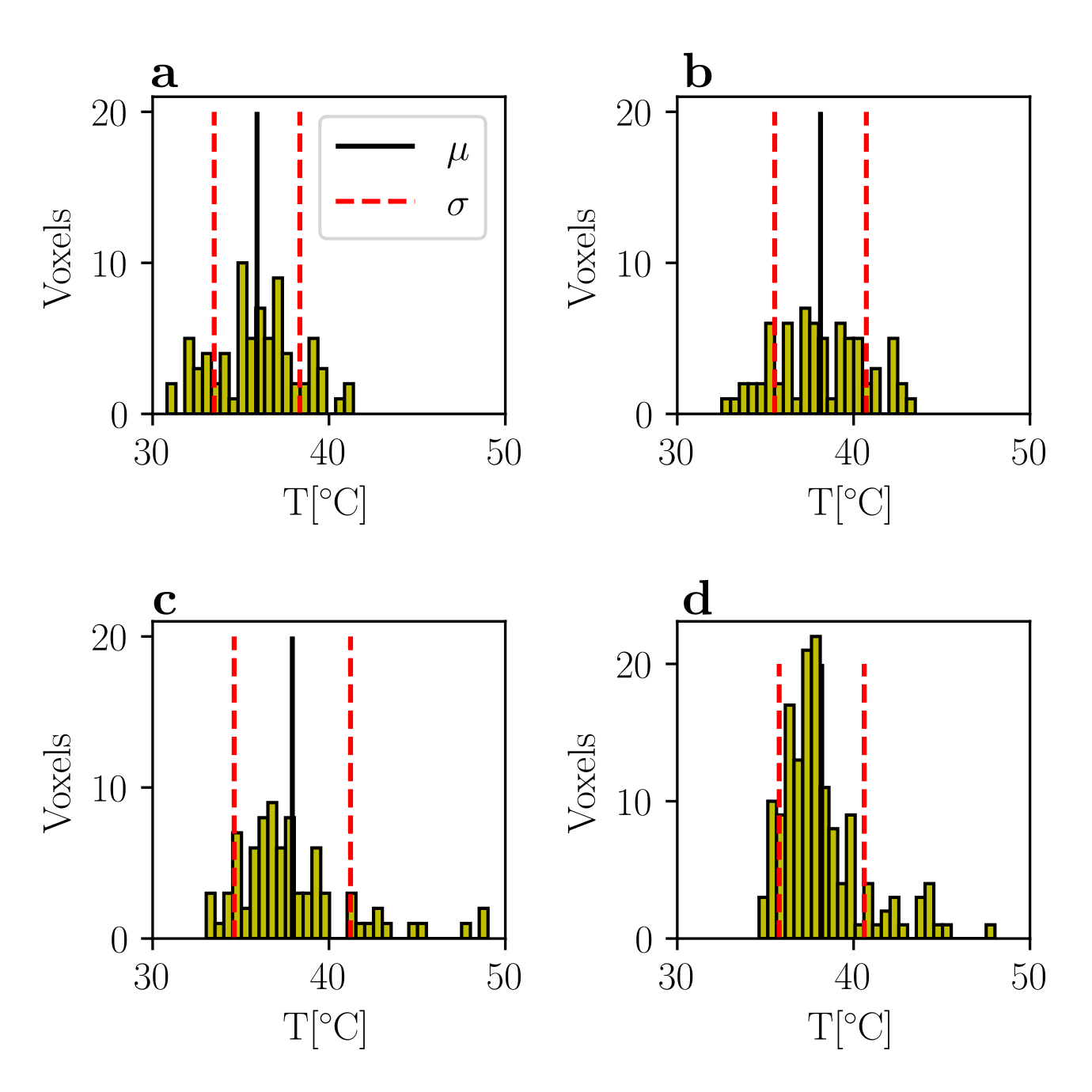


**Figure S10: Apparent temperature map histograms for mouse brain CSI data.** Animal 1 had an overall apparent temperature of 35.9±2.4 °C (panel a, n=76) and 38.1±2.6°C (panel b, n=71). For Animal 2, the apparent temperature was 37.9±3.3 °C (panel c, n=81) and 38.2±2.4 °C (panel d, n=149). Rectal temperature for respective experiments in a-d was: 37.2±0.1 °C, 38.2±0.1 °C, 37.8±0.1 °C and 37.7±0.1 °C.

## Influence of SNR, line width, sampling and line broadening on apparent temperature estimation


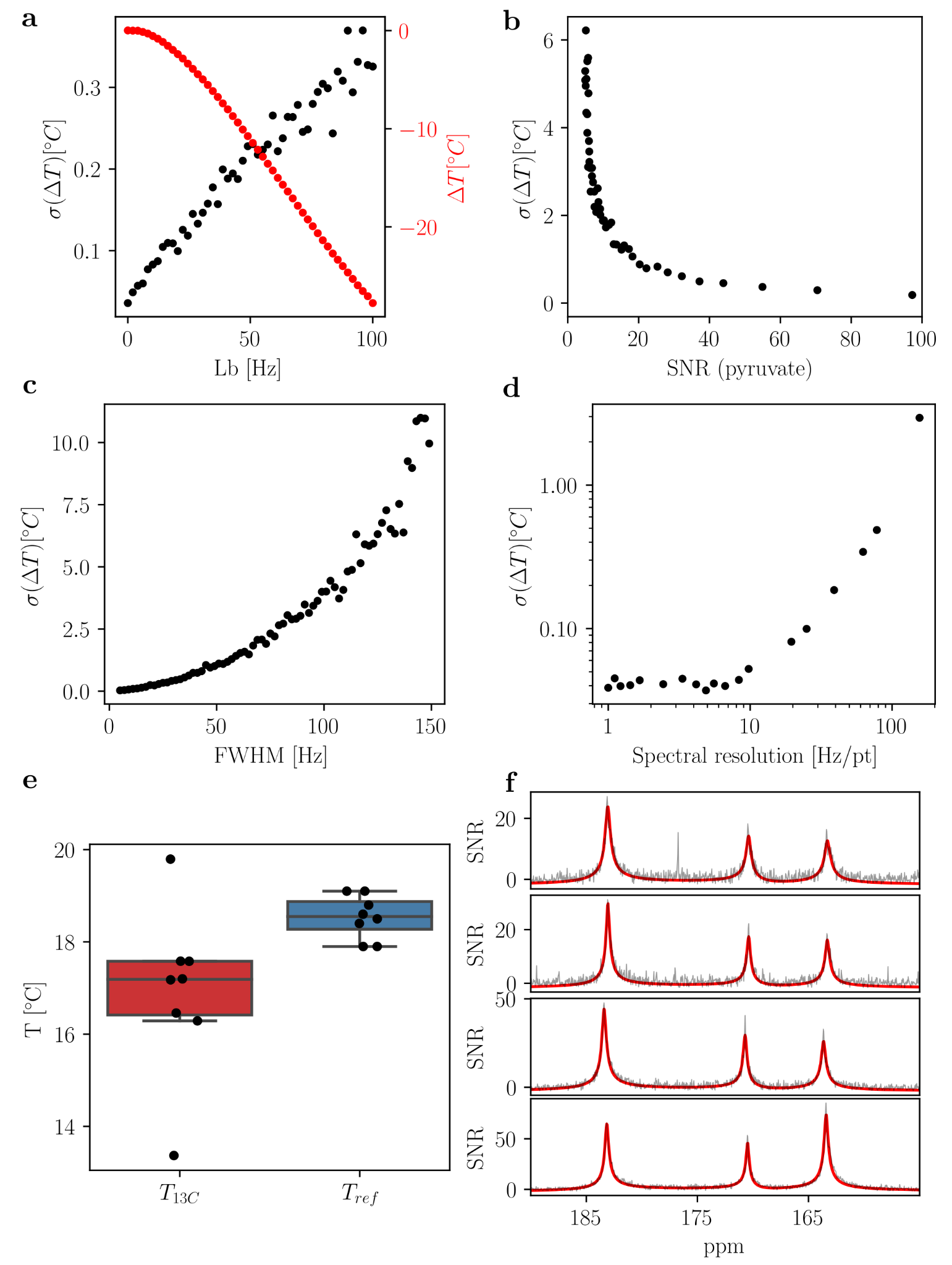
**Figure S11: Influence of line broadening, SNR, line width and spectral resolution on apparent temperature measurements.**

a: Simulated artificial spectra with pyruvate, lactate, pyruvate-hydrate and bicarbonate peaks were fitted with varying line broadening (50 values from 0-100 Hz, repeated each 100 times). In black, the standard deviation of the difference between true and fitted temperature is shown, which increases with increasing line broadening. In red the difference between fitted and true temperature, showcasing, that line broadening leads to an lower apparent temperatures by moving peaks closer together. b: Effect of peak SNR on fitting accuracy (50 noise levels), standard deviation over 100 repetitions of true vs fitted temperature decreases for increasing SNR as expected. c: Effect of line width on fitting accuracy. Spectra with line width from 5-150 Hz were generated and fitted. Standard deviation over 100 repetitions of true vs fitted temperature increases for increasing line width as expected. d: Effects of spectra resolution on fitting accuracy. e: When compared to low SNR spectra acquired for blood-oxygenation experiments described in section 4, Figure S7, the apparent temperature from ^13^C agree within the uncertainties (mean: 16.9±1.8 °C vs 18.5±0.1 °C, lines in boxplot are median and whiskers are quartiles) with a reference measurement using a Pt100 probe. Additionally, a 200 mM concentration curve was used, while the samples had a concentration of 220-240 mM ^13^C. Using a 200 mM curve is expected to lead to slightly lower apparent temperatures, if the true concentration is a little bit higher, especially in this regime, see Figure 1. f: Four exemplary spectra and fits used for e).

Simulation parameters: bandwidth=5000 Hz, frequencies=19.0, 410.0, -313.0, 273.0 Hz, field=3 T, T2*=50 ms (except for variation of line width simulation in S10c, there: T2*=2.1-63 ms), relative peak heights=1.2, 0.85, 0.3, 0.3 , sampling points=2048 (except for variation of spectral resolution in S10d, there: points=32-5000), each simulation was repeated 100 times for statistics.

# References

1. Nagel, L. et al. Parahydrogen-Polarized [1-13C]Pyruvate for Reliable and Fast Preclinical Metabolic Magnetic Resonance Imaging (Adv. Sci. 30/2023). Adv. Sci. 10, 2370208 (2023).

2. Skinner, J. G. et al. Spectrally selective bSSFP using off‐resonant RF excitations permits high spatiotemporal resolution 3D metabolic imaging of hyperpolarized [ 1‐ 13 C ]Pyruvate‐to‐[ 1‐ 13 C ]lactate conversion. Magn. Reson. Med. 90, 894–909 (2023).

3. Kaggie, J. D. et al. Deuterium metabolic imaging and hyperpolarized 13C-MRI of the normal human brain at clinical field strength reveals differential cerebral metabolism. NeuroImage 257, 119284 (2022).

4. Zaccagna, F. et al. Imaging Glioblastoma Metabolism by Using Hyperpolarized [1- 13 C]Pyruvate Demonstrates Heterogeneity in Lactate Labeling: A Proof of Principle Study. Radiol. Imaging Cancer 4, e210076 (2022).

5. Ma, J. et al. Dynamic 13C MR spectroscopy as an alternative to imaging for assessing cerebral metabolism using hyperpolarized pyruvate in humans. Magn. Reson. Med. 87, 1136–1149 (2022).

6. Ursprung, S. et al. Hyperpolarized 13C-Pyruvate Metabolism as a Surrogate for Tumor Grade and Poor Outcome in Renal Cell Carcinoma—A Proof of Principle Study. Cancers 14, 335 (2022).

7. Wolber, J., Cherubini, A., Dzik-Jurasz, A. S. K., Leach, M. O. & Bifone, A. Spin-lattice relaxation of laser-polarized xenon in human blood. Proc. Natl. Acad. Sci. 96, 3664–3669 (1999).

8. Merino, C. Formulas for Temperature PaO2 Correction. Anesth. Analg. 89, 1065 (1999).

9. Hansen, D., Syben, R., Vargas, O., Spies, C. & Welte, M. The Alveolar-Arterial Difference in Oxygen Tension Increases with Temperature-Corrected Determination During Moderate Hypothermia. Anesth. Analg. 88, 538 (1999).
